# Air-to-Air Optical Wireless Communication for Small Fixed-Wing UAVs: Jitter Characterization, System Integration, and Flight Trials

**DOI:** 10.3390/s26216896

**Published:** 2026-10-30

**Authors:** Wentao Li, Yongchun Yuan, Zijian Yuan, Shulei Wang, Weibo Zheng

**Affiliations:** 1 Shanghai Institute of Technical Physics, Chinese Academy of Sciences, Shanghai 200083, China; liwentao21@mails.ucas.ac.cn (W.L.); yuanyongchun@mail.sitp.ac.cn (Y.Y.); yuanzijian23@mails.ucas.ac.cn (Z.Y.); 2 University of Chinese Academy of Sciences, Beijing 100049, China; 3 Key Laboratory of Specialty Optics and Optical Fiber Access Networks, Shanghai University, Shanghai 200444, China; wangshulei@shu.edu.cn

**Keywords:** optical wireless communication, unmanned aerial vehicles, platform jitter, pointing misalignment, air-to-air flight trials, small fixed-wing UAVs

## Abstract

Applying optical wireless communication (OWC) to small fixed-wing unmanned aerial vehicles (UAVs) is challenging due to pointing misalignment and strict size, weight, and power (SWaP) constraints. To analyze these constraints, we combine platform characterization with a custom flight attitude dataset, model UAV flight jitter from flight telemetry using a location-scale *t*-distribution, and derive a semi-analytical expression for the average bit error rate (BER) of inter-UAV OWC links under Gamma-Gamma turbulence. Furthermore, we propose an inter-UAV OWC scheme that combines a wide-angle optical transceiver for passive pointing tolerance, feedforward pointing compensation to mitigate optical-axis misalignment caused by continuous relative motion and rapid maneuvers, and rate adaptation to improve transmission reliability under dynamic channel fading; flight-log-driven simulations evaluate the feedforward tracking performance. Airborne flight trials validate full-duplex communication and three-node relaying among small fixed-wing UAVs, demonstrating real-time video transmission at 50 Mbps over a link distance exceeding 100 m and a three-UAV relay network at 20 Mbps over a cumulative range exceeding 300 m under dynamic cruising conditions.

## 1. Introduction

**Figure 1:**
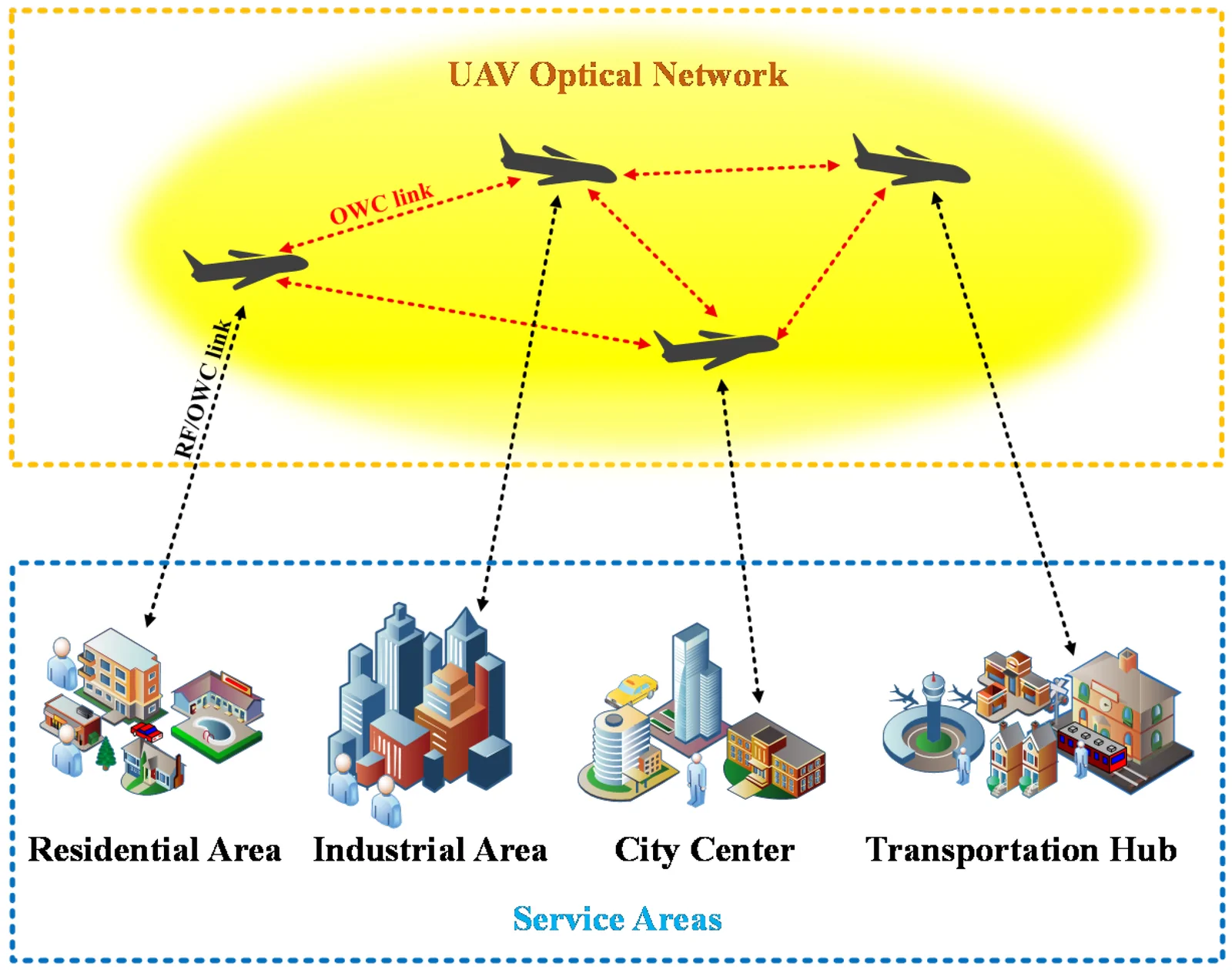
Deployment and application scenario of inter-UAV OWC networks.

Intelligent unmanned aerial vehicle (UAV) swarms represent a pivotal advancement in autonomous systems, requiring robust inter-node communication for formation control, collaborative sensing, and distributed decision-making. Recent advances in UAV-assisted wireless networks have explored joint clustering, transmission, and trajectory design to improve information freshness, highlighting the importance of timely data delivery in aerial networks [1]. Currently, inter-UAV communication relies heavily on radio frequency (RF) links. However, RF systems face challenges such as strict spectrum regulation, limited bandwidth, and vulnerability to jamming. Optical wireless communication (OWC) is a high-bandwidth, secure alternative offering large data rates and high immunity to electromagnetic interference [2]. Establishing inter-UAV OWC mesh networks is therefore a promising approach for high-speed, secure aerial networking. Figure 1 illustrates a typical deployment scenario of an inter-UAV OWC network in an urban environment, providing high-speed optical links to interconnect different regions.

Nonetheless, deploying OWC on small fixed-wing UAVs faces three primary challenges [2,3,4,5,6,7,8,9]:

1. Channel fading: Atmospheric turbulence and high-speed platform mobility induce severe, time-varying channel attenuation.
2. Pointing misalignment: Aerodynamic turbulence and platform maneuvering cause attitude jitter, disrupting transceiver alignment.
3. Strict size, weight, and power (SWaP) constraints: Small UAVs have highly restricted payload weight, volume, and power capacities, limiting the deployment of active tracking systems.

Building on foundational BER analysis of turbulence and pointing errors [10], recent studies have investigated UAV-specific channel impairments and their mitigation [3,7,11,12,13,14]. Specifically, Najafi et al. [9] developed geometric and misalignment loss models for hovering UAVs with position and orientation fluctuations and analyzed outage probability and ergodic rate. Ghanbari et al. [8] optimized beamwidth and flight altitude for UAV-to-HAP FSO links under pointing inaccuracies and beam wandering. Moon et al. [11] incorporated roll, pitch, and yaw jitter into a fixed-wing UAV pointing-error model and applied it to energy-efficient trajectory optimization for UAV-to-ground links. These studies establish the importance of platform-dependent pointing statistics; however, hovering-platform models and fixed-wing air-to-ground trajectory analyses do not directly characterize the measured attitude fluctuations and link geometry of two continuously moving fixed-wing UAVs.

On the hardware side, existing approaches maintain optical connectivity through mechanical tracking, enlarged angular coverage, or retroreflective architectures. Khan et al. [15] developed GPS-free alignment protocols using mechanically steerable transceivers on autonomous mobile nodes, with simulation and prototype validation. Chang et al. [16] employed a light-diffusing-fiber transmitter and camera-based decoding to provide circumferential optical coverage. These approaches address active alignment and angular coverage, respectively, but their reported configurations do not establish full-duplex inter-UAV operation during fixed-wing cruising. Alternatively, modulating retro-reflectors (MRRs) reduce the airborne terminal burden by modulating an externally supplied optical beam [17]. Quintana et al. [18] demonstrated a UAV-to-ground MRR link at 500 Mbps over 560 m, with a BER of $7.6\times 10^{-4}$. Although this architecture reduces the need for an onboard optical transmitter, it relies on external illumination and a return optical path, differing from independently transmitting inter-UAV terminals. Beyond these approaches, Pan et al. [19] explored joint deployment, user association, and power optimization for UAV-mounted STAR-RIS-assisted maritime links. Although developed for RF communications, this approach offers a potential design perspective for future reconfigurable optical links.

Existing airborne demonstrations provide complementary advances in optical localization, mobile alignment, angular coverage, and transmission capacity. Horyna et al. [20] integrated communication based on blinking UV markers and onboard cameras with relative localization, enabling short-range information exchange for cooperative UAV operation. Liang et al. [21] combined image identification, a three-axis gimbal, and white-light transceivers to demonstrate 2 Mbps mobile full-duplex communication in air-to-ground and air-to-air scenarios; their network-quality measurements identified a 13.1 m distance boundary below which packet loss fell below 5% as the terminals approached from longer ranges. Lee et al. [22] demonstrated a fiber-tethered inter-UAV optical link at up to 2 Gbps over 2 m during indoor hovering, using offline digital signal processing; this controlled proof of concept did not evaluate outdoor propagation or flight-dynamic effects. Li et al. [23] developed an azimuthally omnidirectional annular compound parabolic concentrator (ACPC) receiver with a 114° field of view and demonstrated outdoor image transmission at 9.6 kbps between UAVs approximately 6 m apart. These results demonstrate distinct capabilities, but do not establish the combined capability of active full-duplex communication and multi-UAV relaying during continuous fixed-wing cruising. Table 1 summarizes the experimental conditions and performance of these systems alongside our work.

To address these gaps, we propose a SWaP-compliant, misalignment-tolerant inter-UAV OWC scheme featuring a two-layer link maintenance strategy. Our primary contributions are:

(1) We characterize attitude jitter using measured fixed-wing flight telemetry and derive a semi-analytical average BER expression for inter-UAV OWC links under location-scale *t*-distributed pointing errors and Gamma-Gamma turbulence. Complementing hovering-drone geometric-loss modeling and fixed-wing air-to-ground pointing and trajectory analysis, this contribution connects measured flight-jitter statistics with the performance analysis of inter-UAV optical links.
(2) We adapt and integrate the wide-angle NIR-OWC transceiver architecture established in our prior work [26] onto small fixed-wing UAV platforms. The contribution here is the platform-specific airborne integration and flight validation of this hardware foundation under UAV payload constraints.
(3) We propose a two-layer link maintenance strategy that complements passive pointing tolerance with flight-telemetry-based feedforward pointing compensation and rate-adaptive feedback protection. Feedforward tracking performance is evaluated through flight-log-driven simulations; automated rate adaptation is proposed but was not implemented in the flight trials.
(4) We demonstrate full-duplex OWC and three-UAV relaying on small fixed-wing platforms during continuous forward flight. Moving beyond recent demonstrations with rotary-wing UAVs or under indoor hovering conditions, we establish inter-UAV optical links during sustained fixed-wing cruise, achieving full-duplex video transmission at 50 Mbps over a link distance exceeding 100 m, as well as three-UAV relaying at 20 Mbps over a cumulative range exceeding 300 m. The two relay hops span 126.5 m and 345.0 m, respectively, yielding a cumulative three-dimensional link distance of 471.5 m.

The remainder of this paper is organized as follows: Section 2 details the platform jitter characterization and channel fading model; Section 3 describes the misalignment-tolerant transceiver and link maintenance design; Section 4 presents the experimental platform and flight trial results; and Section 5 concludes the paper.

## 2. Platform Jitter Characterization and Air-to-Air Channel Modeling

### 2.1. Fixed-Wing UAV Platform Constraints and OWC Challenges

Unlike rotary-wing platforms, fixed-wing UAVs must maintain forward motion to generate aerodynamic lift, making them suitable for long-range, high-speed, and wide-area missions. However, the lack of hovering capability and the dynamic nature of swarm formations result in continuous variations in relative distance and orientation. Furthermore, aerodynamic turbulence and control maneuvers induce continuous small-amplitude attitude jitter. These factors cause rapid received optical power fluctuations, presenting unique challenges for link maintenance. The primary platform characteristics governing OWC deployment include:

(1) High-speed node mobility. Fixed-wing UAVs cruise at higher speeds than rotary-wing platforms, which complicates link tracking and increases the rate of topology changes [27].
(2) Dynamic node topology. Swarm reconfigurations and task reassignments continuously change relative distances, azimuths, and elevations, requiring rapid gimbal tracking.
(3) Platform jitter. Aerodynamic turbulence and maneuvering commands cause roll, pitch, and yaw jitter, perturbing the transceiver alignment.
(4) Strict SWaP constraints. Small fixed-wing UAVs have highly restricted payload weight, volume, and power capacities, demanding integrated, low-power OWC designs.

These platform constraints dictate that deploying OWC systems on fixed-wing UAVs requires balancing transceiver alignment bandwidth, beam divergence, and receiver gain. Unlike static ground-based links, the airborne channel is dominated by rapid scintillation and motion-induced pointing errors, necessitating robust physical design and active tracking.

### 2.2. UAV Flight Jitter Characteristics

**Figure 2:**
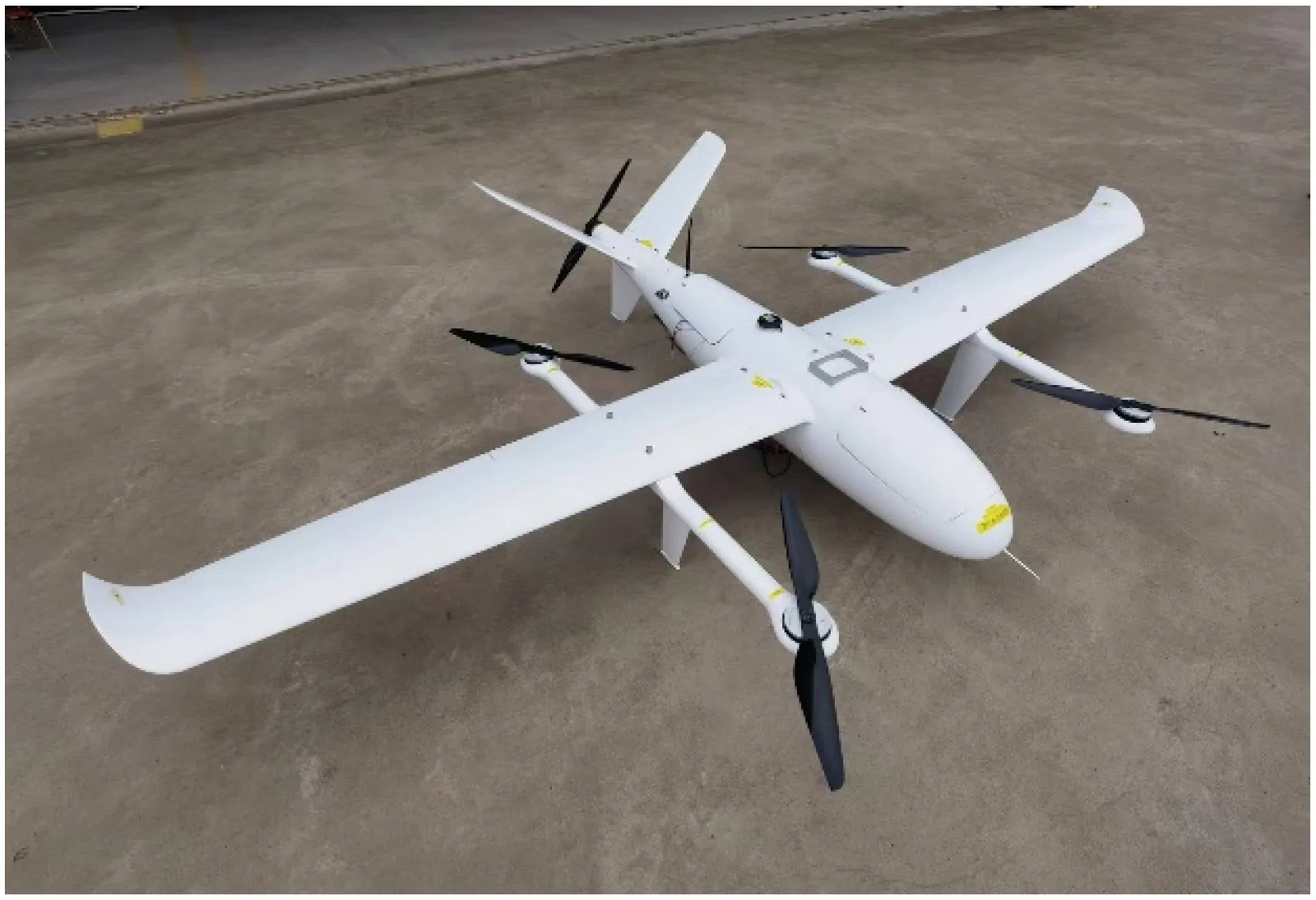
The INNNO ING-3500 used in the flight trials.

To characterize fixed-wing UAV attitude jitter under real-world conditions, we collected telemetry logs from flight experiments to establish a flight attitude dataset. We utilized the INNNO ING-3500 vertical takeoff and landing (VTOL) fixed-wing UAV (Figure 2), whose specifications are listed in Table 2. The dataset includes 15 sorties (nearly 700 min, approximately 600 km cumulative range) covering single-UAV, two-UAV, and three-UAV formations. Available telemetry includes GPS position, altitude, airspeed, ground speed, wind speed, and 3D attitude angles. Table 3 summarizes the cruise-phase flight parameters, single flight range, and duration. In Table 3, altitude is obtained from the logged relative-altitude field, ground speed and airspeed are converted from the corresponding telemetry fields to m/s, and wind speed is calculated as the magnitude of the horizontal EKF wind vector. Their minimum, maximum, and mean are calculated over cruise samples pooled across all 15 sorties. For each flight record, distance is estimated by integrating ground speed at the nominal 50 Hz sampling rate, and duration is calculated as the sample count divided by 50. The minimum, maximum, and mean of these per-sortie estimates are reported in the final two rows. The dataset is publicly available at https://github.com/LiWentao0316/Fixed-Wing-UAV-Attitude-Dataset (accessed on 11 October 2026).

**Figure 3:**
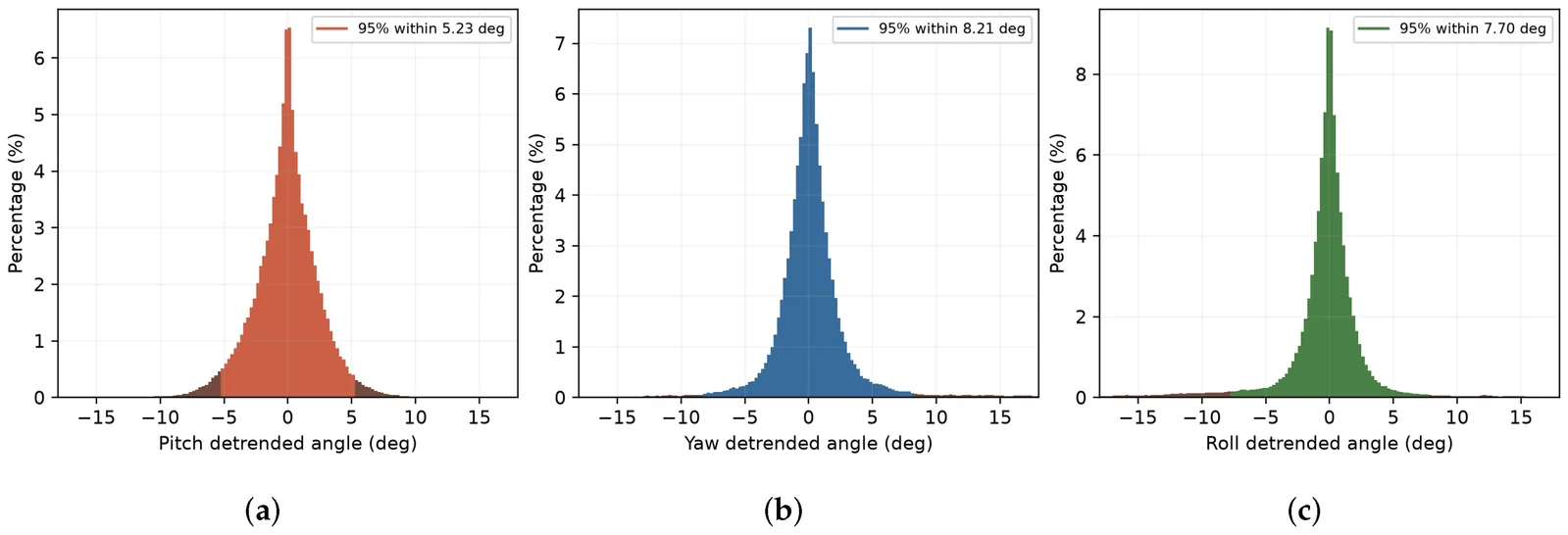
Empirical distribution statistics of detrended attitude angle variations during straight flight cruise across all 15 flight trials under unified coordinate axes: (**a**) pitch, (**b**) yaw, and (**c**) roll.

Jitter variations during straight cruise were isolated using speed and commanded attitude records, followed by detrending with a 500-sample moving mean. For each sortie and attitude axis, residuals exceeding the 99.5% quantile of the absolute detrended angle were excluded. The processed 3D attitude fluctuations are shown in Figure 3.

Statistical analysis shows that the pitch, yaw, and roll fluctuations during straight cruise follow zero-mean, bell-shaped distributions. The empirical dataset comprises 707,823 discrete attitude telemetry samples across 15 flight trials (corresponding to approximately 236 min of steady straight cruise). Here, σ denotes the sample standard deviation (expressed in degrees, with $\sigma^{2}$ representing the variance). In Figure 3, all three subfigures share an identical horizontal axis range of $\left[-18^{\circ },18^{\circ }\right]$ for fair comparison. The pitch angle (a) is the most stable ($\sigma =2.50^{\circ }$, 95% within $5.23^{\circ }$), while the roll (c) ($\sigma =3.33^{\circ }$, 95% within $7.70^{\circ }$) and yaw (b) ($\sigma =5.40^{\circ }$, 95% within $8.21^{\circ }$) exhibit larger variations, identifying yaw as the primary jitter source.

To investigate the physical drivers of attitude disturbances, Table 4 evaluates the linear association between the per-flight attitude standard deviations and environmental parameters across the 15 flight trials using the Pearson correlation coefficient *r* and the associated two-tailed *p*-value:

(1) $$r=\frac{\sum_{i=1}^{n}\left(X_{i}-\bar{X}\right)\left(Y_{i}-\bar{Y}\right)}{\sqrt{\sum_{i=1}^{n}{\left(X_{i}-\bar{X}\right)}^{2}}\sqrt{\sum_{i=1}^{n}{\left(Y_{i}-\bar{Y}\right)}^{2}}},\;t=r\sqrt{\frac{n-2}{1-r^{2}}}$$

where n=15 (degrees of freedom df=13), and a significance threshold of p<0.05 denotes statistical significance. The aerodynamic mechanisms underlying these correlations are interpreted as follows:

1. Pitch jitter mechanism: Airspeed standard deviation (r=0.521,p=0.047) and mean acceleration (r=−0.568,p=0.027) correlate significantly with pitch jitter. Because aerodynamic lift $L_{\mathrm{aero}}=\frac{1}{2}\rho v^{2}SC_{L}$ depends quadratically on airspeed, rapid airspeed fluctuations induce dynamic lift-gravity imbalances, exciting longitudinal short-period pitching oscillations. Concurrently, longitudinal acceleration variations introduce inertial pitching moments that further exacerbate pitch jitter.
2. Yaw jitter mechanism: Mean wind speed (r=0.647,p=0.009) and wind speed standard deviation (r=0.602,p=0.017) exhibit strong, statistically significant correlations with yaw jitter. Crosswind components directly exert aerodynamic side-forces on the vertical tailplane (the weathercock stability effect), rendering yaw jitter particularly sensitive to ambient wind speed and atmospheric turbulence.
3. Roll jitter mechanism: Mean wind speed also shows a significant positive correlation with roll jitter (r=0.535,p=0.040). Crosswinds passing over high-aspect-ratio wings generate asymmetric local angles of attack and differential lift across the left and right wings, generating rolling moments that degrade lateral stability.

Additionally, while researchers commonly model UAV jitter using a normal distribution, this assumption may be inaccurate in practice [11,28]. By evaluating the Kolmogorov-Smirnov (KS) test statistics and Kullback-Leibler (KL) divergence values presented in Table 5, we find that the empirical distribution is closest to the Location-Scale *t*-distribution overall. Although the Laplace distribution yields slightly lower test statistics for the pitch angle alone, the Location-Scale *t*-distribution demonstrates an overwhelming advantage across the other two dominant jitter dimensions. Consequently, the *t*-distribution achieves the best comprehensive average fit across all three axes. Furthermore, choosing the unified Location-Scale *t*-distribution family across all three attitude angles ensures mathematical consistency, facilitating subsequent joint pointing error modeling and channel derivations. In contrast, the widely used Normal distribution performs poorly across the dimensional comparisons: in the KS test, it only slightly outperforms the Cauchy distribution for the pitch angle while ranking last in the other two dimensions; in terms of KL divergence, the Normal distribution gives the largest discrepancy for yaw and roll. To explain this non-Gaussian heavy-tailed behavior, two tentative hypotheses are proposed:

1. Atmospheric turbulence intermittency: Atmospheric boundary-layer measurements show that turbulent velocity increments can exhibit intermittent, non-Gaussian statistics [29]. Occasional large wind increments can generate transient aerodynamic loads, which may contribute to infrequent attitude excursions beyond those predicted by a Gaussian model. In our dataset, the correlations of yaw jitter with mean wind speed and wind-speed variability (Table 4) provide indirect support for a wind-related contribution. It should be noted, however, that these correlations do not establish turbulence intermittency as the cause of the observed heavy tails.
2. Control-system nonlinearities and possible actuator saturation: The attitude response depends on the closed-loop interaction between aerodynamic disturbances and the flight controller. Finite actuator deflection and rate limits may restrict disturbance rejection during strong gusts, allowing larger transient attitude errors. The anti-windup control study in [30] demonstrates the relevance of input saturation to UAV attitude control. However, actuator saturation has not been verified in the present flight records and therefore remains a tentative hypothesis.

It should be noted that the location-scale *t*-distribution is adopted on the basis of the empirical goodness-of-fit results, rather than on these mechanistic hypotheses. The two mechanisms are proposed only as possible explanations for the observed heavy-tailed behavior.

Nevertheless, it is worth noting that even the Location-Scale *t*-distribution is still insufficient to fully characterize the attitude jitter characteristics.

Table 6 reports the specific estimated parameters of the five candidate distributions across the pitch, yaw, and roll attitude axes. The primary model parameters presented in the table were estimated via maximum likelihood estimation (MLE) over the entire steady-state straight cruise dataset (N=707,823 discrete samples across all 15 flight sorties, representing approximately 236 min of steady straight cruise flight). This aggregated estimation captures the complete operational envelope across varying flight sorties and wind conditions. For the optimal Location-Scale *t*-distribution, the estimated location parameters μ along all three axes remain strictly close to zero ($\left|\mu \right|\leq 0.056^{\circ }$). The degrees of freedom ν for yaw ($\nu_{y}=1.42$) and roll ($\nu_{r}=1.42$) are notably lower than 2, confirming prominent heavy-tailed non-Gaussian traits, whereas pitch exhibits a higher degree of freedom ($\nu_{p}=5.03$) reflecting tighter longitudinal elevator control. Crucially, the scale parameter σ of the *t*-distribution must be distinguished from the empirical standard deviation: for heavy-tailed regimes with ν≤2, theoretical variance and standard deviation are mathematically undefined (infinite), so the scale parameter σ serves as the well-defined dispersion metric characterizing the distribution’s central spread.

To examine between-sortie variability, supplementary maximum-likelihood fits were obtained for each sortie after detrending and 99.5% quantile pruning. Mean straight-cruise wind speeds range from 1.30 to 7.08 m/s. Across the 15 sorties, the pitch, yaw, and roll scale parameters range from $0.14^{\circ }$ to $3.33^{\circ }$, $0.29^{\circ }$ to $2.33^{\circ }$, and $0.12^{\circ }$ to $2.43^{\circ }$, respectively. Median degrees of freedom are 12.28, 1.73, and 1.85; yaw and roll have ν<2 in 11 and 10 sorties, respectively, whereas some pitch fits approach the Gaussian limit. Thus, pronounced heavy tails recur across multiple sorties, particularly in yaw and roll, but neither the tail index nor the scale is invariant across flights. No monotonic association between mean wind speed and fitted scale is detected in this sample (Spearman $|r_{s}|\leq 0.26$, p>0.35 for all axes); this does not exclude effects of wind variability or other flight conditions. The pooled parameters characterize the aggregate dataset rather than a universal per-sortie distribution.

Another point worth noting is the use of 99.5% quantile pruning in the data preprocessing, which removes the largest 0.5% of absolute detrended attitude deviations in each sortie. This may raise concerns about bias when fitting a heavy-tailed location-scale *t* distribution. To examine this effect, we evaluated the KS goodness of fit for the data before and after pruning, fitting the distribution separately in each case. The overall KS goodness-of-fit statistics change only slightly before and after pruning, indicating a limited effect on the overall distribution fit. The KS statistic increases slightly from 0.02315 to 0.02464 for pitch, decreases from 0.01958 to 0.01702 for yaw, and remains nearly unchanged for roll, from 0.02164 to 0.02138. Thus, the overall agreement between the fitted distributions and their respective empirical data remains similar. Nevertheless, amplitude-based pruning inevitably shifts empirical tail quantiles toward smaller absolute deviations and can introduce substantial errors in extreme-tail analysis.

### 2.3. Air-to-Air OWC Channel Model with Jitter-Induced Pointing Errors

The inter-UAV OWC channel attenuation is composed of geometric loss and atmospheric turbulence. For the reported clear-air trials, small deterministic atmospheric losses are neglected as an engineering approximation; the visibility-based estimate and solar-background noise budget are discussed in Section 3.2. Platform motion introduces pointing errors, while atmospheric turbulence causes additional irradiance fluctuations. Thus, our channel model focuses on pointing errors and atmospheric turbulence.

For an on-off keying (OOK) modulated OWC system, the received signal is modeled as y=hx+n, where x∈{0,a} is the transmitted optical signal power with amplitude *a*, and $n∼N(0,\sigma^{2})$ is additive white Gaussian noise. The channel attenuation coefficient *h* is factored as $h=h_{g}h_{a}$, where $h_{g}$ and $h_{a}$ represent the pointing-error-induced geometric loss and the atmospheric-turbulence-induced intensity scintillation, respectively.

**Figure 4:**
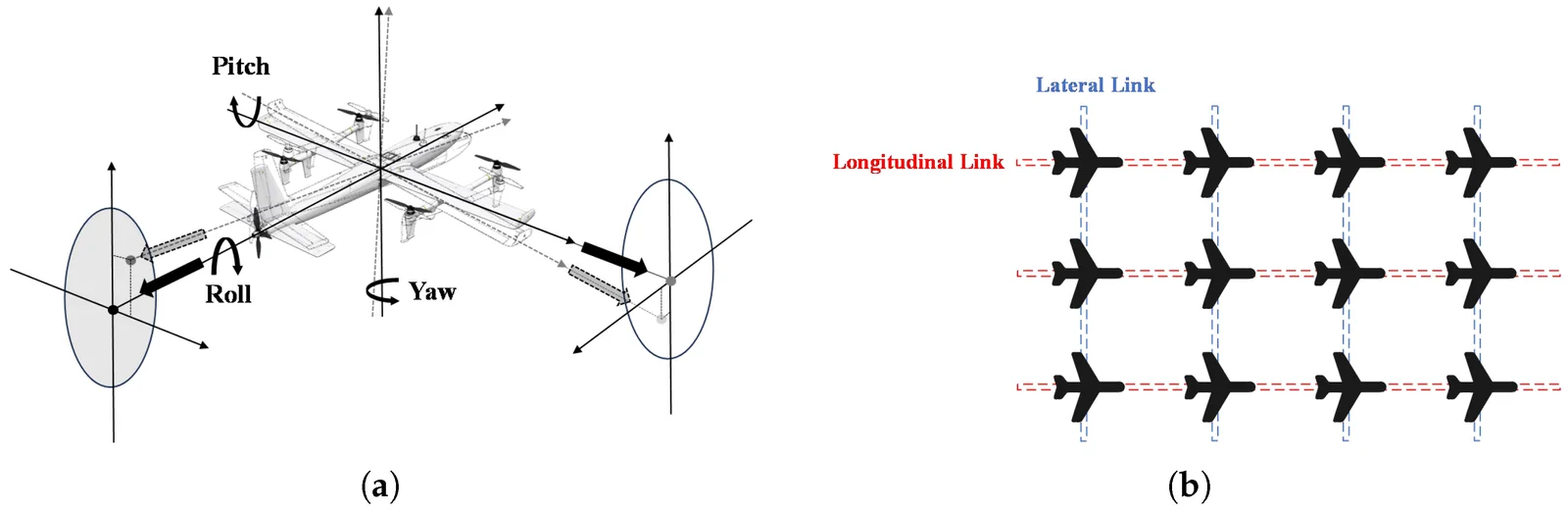
Schematic diagrams of the inter-UAV optical link: (**a**) influence of UAV flight jitter on links in different directions, and (**b**) rectangular mesh formation topology of UAVs.

Geometric attenuation depends on the beam divergence, receiver orientation, range, detector aperture, and beam profile. For mid-range links, vertical-cavity surface-emitting lasers (VCSELs) with Gaussian beam profiles are commonly utilized. Assuming initial alignment, the pointing error $\theta_{t}$ is typically within $10^{\circ }$, and under the small-angle approximation, the geometric attenuation coefficient $h_{g}$ at the receiver is expressed as [26]:

(2) $$h_{g}\approx A_{0}\exp\left(-\frac{\ln2\cdot \theta_{t}^{2}}{\tan^{2}\left(\theta_{\mathrm{HPHA}}\right)}\right)$$

(3) $$A_{0}=\frac{\ln2\cdot A_{d}\cos\left(\omega_{r}\right)}{\pi L^{2}\tan^{2}\left(\theta_{\mathrm{HPHA}}\right)}$$

where *L* is the inter-UAV optical wireless transmission distance (link range), $A_{d}$ is the receiver aperture area, $\omega_{r}$ is the angle of arrival, and $\theta_{\mathrm{HPHA}}$ is the half-power half-angle (HPHA) of the transmitter beam. In this air-to-air geometric model, attitude jitter from both UAV platforms is incorporated: fluctuations of the transmitting UAV deflect the optical beam axis relative to the line of sight (LOS), defining the transmitter pointing error $\theta_{t}$, while fluctuations of the receiving UAV perturb the receiver orientation, altering the angle of arrival $\omega_{r}$. Moreover, the receiver possesses a finite optical field of view (FOV), $\theta_{\mathrm{FOV}}$. Within the FOV ($\omega_{r}\leq \theta_{\mathrm{FOV}}/2$), the effective aperture area scales with $\cos(\omega_{r})$, whereas outside the FOV ($\omega_{r}>\theta_{\mathrm{FOV}}/2$), optical collection is sharply cut off. For the wide-angle receiver optics operating under steady cruise jitter (≤$10^{\circ }$), $\omega_{r}$ remains well within the FOV cutoff with $\cos(\omega_{r})\approx 1$. Pointing fluctuations are highly dependent on the link direction: longitudinal links are dominated by pitch and yaw, whereas lateral links are primarily perturbed by roll and yaw, as shown in Figure 4a.

In a complex UAV swarm flight, multiple link states typically coexist, and pointing deviations in certain link configurations—such as a triangular formation—can even be simultaneously affected by all three attitude angles. For ease of analysis, the rectangular mesh formation shown in Figure 4b is adopted as the basis for subsequent discussions. This formation contains longitudinal and lateral links, both of which are only related to two directional attitude angles. Based on the previous analysis, the empirical distribution of individual attitude angles approaches the location-scale *t*-distribution. Consequently, we assume that the pointing errors in the two tracking axes, $\theta_{x}$ and $\theta_{y}$, are independent, zero-mean location-scale *t*-distributed random variables with shape parameters (degrees of freedom) $v_{x}$, $v_{y}$ and scale parameters $\sigma_{x}$, $\sigma_{y}$. This assumed statistical independence between the orthogonal tracking axes is physically grounded in the fact that longitudinal dynamics (pitch, driven by elevators) and lateral-directional dynamics (yaw/roll, driven by rudders/ailerons) of fixed-wing aircraft are aerodynamically decoupled to first order during steady cruise, and it is corroborated by the mechanical orthogonality of the 2-axis gimbal actuators and the negligible cross-correlation (|r|<0.12) observed in our flight telemetry dataset. This decoupling convention enables tractable derivation of the composite radial pointing error:

(4) $$\theta_{x}∼t_{\mathrm{ls}}\left(0,\sigma_{x}^{2},v_{x}\right),\;\theta_{y}∼t_{\mathrm{ls}}\left(0,\sigma_{y}^{2},v_{y}\right)$$

where $t_{\mathrm{ls}}\left(\mu ,\sigma^{2},v\right)$ denotes the three-parameter Location-Scale Student’s *t*-distribution, with location parameter μ=0, scale parameters $\sigma_{x},\sigma_{y}$, and degrees-of-freedom shape parameters $v_{x},v_{y}$. The radial pointing error is $\theta_{t}=\sqrt{\theta_{x}^{2}+\theta_{y}^{2}}$. The probability density function (PDF) of $\theta_{t}$ is derived in Appendix A as follows:

(5) $$f_{\theta_{t}}\left(\theta \right)=A\theta {\left(1+\frac{\theta^{2}}{v_{x}\sigma_{x}^{2}}\right)}^{-\frac{v_{x}+1}{2}}F_{1}\left(\frac{1}{2};\frac{v_{x}+1}{2},\frac{v_{y}+1}{2};1;\frac{\theta^{2}}{v_{x}\sigma_{x}^{2}+\theta^{2}},-\frac{\theta^{2}}{v_{y}\sigma_{y}^{2}}\right)$$

where $F_{1}$ is the Appell hypergeometric function, and

(6) $$A=\frac{2\Gamma \left(\frac{v_{x}+1}{2}\right)\Gamma \left(\frac{v_{y}+1}{2}\right)}{\sigma_{x}\sigma_{y}\sqrt{v_{x}v_{y}}\Gamma \left(\frac{v_{x}}{2}\right)\Gamma \left(\frac{v_{y}}{2}\right)}$$

The radial PDF is obtained by integrating the joint axis-wise density in polar coordinates; the Jacobian supplies the factor θ. With $q=\ln2/\tan^{2}\left(\theta_{\mathrm{HPHA}}\right)$, the geometric-fading transformation uses $\theta =\sqrt{\ln(A_{0}/h_{g})/q}$ and $|d\theta /dh_{g}|={\left(2qh_{g}\theta \right)}^{-1}$. The Pfaff transformation places the Appell arguments within the convergence domain.

By substitution and applying the Pfaff transformation on the second argument of $F_{1}$, where the parameter transforms as follows:

(7) $$b_{2}\to c-b_{1}-b_{2}=1-\frac{v_{x}+1}{2}-\frac{v_{y}+1}{2}=-\frac{v_{x}+v_{y}}{2}$$

to keep the arguments within [0,1), we derive the PDF of the geometric fading $h_{g}$:

(8) $$f_{h_{g}}\left(h_{g}\right)=\frac{BF_{1}\left(\frac{1}{2};\frac{v_{x}+1}{2},-\frac{v_{x}+v_{y}}{2};1;x\left(h_{g}\right),y\left(h_{g}\right)\right)}{h_{g}{\left(1+C\ln\frac{A_{0}}{h_{g}}\right)}^{\frac{v_{x}+1}{2}}{\left(1+D\ln\frac{A_{0}}{h_{g}}\right)}^{\frac{1}{2}}}$$

with

(9) $$x\left(h_{g}\right)=\frac{D\ln\frac{A_{0}}{h_{g}}+CD\ln^{2}\frac{A_{0}}{h_{g}}+C\ln\frac{A_{0}}{h_{g}}}{\left(1+C\ln\frac{A_{0}}{h_{g}}\right)\left(1+D\ln\frac{A_{0}}{h_{g}}\right)},\;y\left(h_{g}\right)=\frac{D\ln\frac{A_{0}}{h_{g}}}{1+D\ln\frac{A_{0}}{h_{g}}}$$

(10) $$B=\frac{\Gamma \left(\frac{v_{x}+1}{2}\right)\Gamma \left(\frac{v_{y}+1}{2}\right)\tan^{2}\left(\theta_{\mathrm{HPHA}}\right)}{\sigma_{x}\sigma_{y}\sqrt{v_{x}v_{y}}\Gamma \left(\frac{v_{x}}{2}\right)\Gamma \left(\frac{v_{y}}{2}\right)\ln2},\;C=\frac{\tan^{2}\left(\theta_{\mathrm{HPHA}}\right)}{v_{x}\sigma_{x}^{2}\ln2},\;D=\frac{\tan^{2}\left(\theta_{\mathrm{HPHA}}\right)}{v_{y}\sigma_{y}^{2}\ln2}$$

The shape parameters in the pointing error distribution govern the tail heaviness (outlier frequency), while the scale parameters determine the jitter dispersion. For atmospheric scintillation, the channel fading $h_{a}$ follows a Gamma-Gamma distribution [31]:

(11) $$f_{h_{a}}\left(h_{a}\right)=\frac{2{\left(\alpha \beta \right)}^{(\alpha +\beta )/2}}{\Gamma (\alpha )\Gamma (\beta )}h_{a}^{(\alpha +\beta )/2-1}K_{\alpha -\beta }\left(2\sqrt{\alpha \beta h_{a}}\right),\;h_{a}>0$$

where $K_{p}\left(x\right)$ is the modified Bessel function of the second kind of order *p*, and the parameters α and β represent the effective number of large-scale and small-scale cells of the scattering process, respectively, which are calculated as follows:

(12) $$\alpha ={\left[\exp\left(\frac{0.49\sigma_{R}^{2}}{{\left(1+1.11\sigma_{R}^{12/5}\right)}^{7/6}}\right)-1\right]}^{-1},\;\beta ={\left[\exp\left(\frac{0.51\sigma_{R}^{2}}{{\left(1+0.69\sigma_{R}^{12/5}\right)}^{5/6}}\right)-1\right]}^{-1}$$

where $\sigma_{R}^{2}$ is the Rytov variance characterizing the channel’s turbulence intensity, given by:

(13) $$\sigma_{R}^{2}=1.23C_{n}^{2}k^{7/6}L^{11/6}$$

where k=2π/λ is the wave number, and the refractive index structure parameter $C_{n}^{2}\left(h\right)$ as a function of altitude is modeled by the Hufnagel-Valley (H-V) 5/7 model [32]:

(14) $$C_{n}^{2}\left(h\right)=\frac{0.00594{\left(v_{\mathrm{HV}}/27\right)}^{2}{\left(10^{-5}h\right)}^{10}}{\exp(h/1000)}+\frac{2.7\times 10^{-16}}{\exp(h/1500)}+\frac{C_{0}}{\exp(h/100)}$$

The turbulence irradiance is normalized so that $E[h_{a}]=1$. The Rytov expression assumes a plane wave and an approximately homogeneous path. In the H–V model, *h* denotes altitude in meters, $v_{\mathrm{HV}}$ is the model wind speed in m/s, and $C_{0}$ is the near-ground structure parameter in $m^{-2/3}$. Assuming independent $h_{g}$ and $h_{a}$, the product-density Jacobian is $1/h_{a}$; the constraint $h_{g}\leq A_{0}$ gives the lower limit $h_{a}\geq h/A_{0}$. This PDF precedes application of the communication boundary.

Combining the distributions of $h_{g}$ and $h_{a}$, the composite channel fading PDF is:

(15) $$f_{h}\left(h\right)=\int_{h/A_{0}}^{\infty }\frac{1}{h_{a}}f_{h_{g}}\left(\frac{h}{h_{a}}\right)f_{h_{a}}\left(h_{a}\right)dh_{a}$$

Using binomial expansion to decouple the arguments of the Appell $F_{1}$ function and integrating, the composite fading PDF $f_{h}\left(h\right)$ is written as a double series-integral representation:

(16) $$f_{h}\left(h\right)=\frac{2B{\left(\alpha \beta \right)}^{(\alpha +\beta )/2}}{\Gamma (\alpha )\Gamma (\beta )h}\sum_{m=0}^{\infty }\sum_{n=0}^{\infty }\Phi \left(m,n\right)\int_{h/A_{0}}^{\infty }\frac{g_{m,n}\left(h/h_{a}\right)K_{\alpha -\beta }\left(2\sqrt{\alpha \beta h_{a}}\right)}{h_{a}^{1-(\alpha +\beta )/2}}dh_{a}$$

where

(17) gm,n(z)=∑j=0mmjCjDm+n−jlnm+nA0z1+ClnA0zj+vx+121+DlnA0zm+n+12

(18) $$\Phi \left(m,n\right)=\frac{{\left(1/2\right)}_{m+n}{\left(\frac{v_{x}+1}{2}\right)}_{m}{\left(-\frac{v_{x}+v_{y}}{2}\right)}_{n}}{{\left(1\right)}_{m+n}m!n!}$$

Expanding $F_{1}$ and applying the binomial theorem gives the double series and the finite sum over *j*. Here, ${\left(a\right)}_{k}=\Gamma \left(a+k\right)/\Gamma \left(a\right)$ is the Pochhammer symbol, and m,n are nonnegative integers.

In Equations (16) and (17), $z=h_{g}=h/h_{a}$. With $u=1/h_{g}=h_{a}/h$, the function becomes $g_{m,n}\left(1/u\right)$ and $\ln\left(A_{0}/z\right)=\ln\left(A_{0}u\right)$. For equiprobable OOK with Gaussian noise variance $\sigma_{N}^{2}$, midpoint detection, and $\mathrm{SNR}=a^{2}/\left(2\sigma_{N}^{2}\right)$, the conditional BER is:

(19) $$P_{e}\left(h\right)=Q\left(h\sqrt{\frac{\mathrm{SNR}}{2}}\right)$$

Using the standard identity:

(20) $$Q\left(z\right)=\frac{1}{2}\mathrm{erfc}\left(\frac{z}{\sqrt{2}}\right)$$

the conditional BER scales to:

(21) $$P_{e}\left(h\right)=\frac{1}{2}\mathrm{erfc}\left(h\sqrt{\frac{\mathrm{SNR}}{4}}\right)$$

The threshold $\theta_{\max}$ is a communication boundary; the underlying *t*-distributed errors remain unbounded. Let $P_{\mathrm{out}}=1-\int_{0}^{\theta_{\max}}f_{\theta_{t}}\left(\theta \right)\;d\theta$. Unavailable-link periods contribute $P_{\mathrm{out}}/2$ to the overall BER under an equiprobable-OOK, no-usable-signal binary-decision convention. The in-boundary integral is therefore not conditionally normalized. The boundary corresponds to $1/A_{0}\leq u\leq u_{\max}$:

(22) $$u_{\max}=\frac{1}{h_{g,\min}}=\frac{1}{A_{0}}\exp\left(\frac{\ln2\cdot \theta_{\max}^{2}}{\tan^{2}\left(\theta_{\mathrm{HPHA}}\right)}\right)$$

Substituting $h_{a}=uh$, with $dh_{a}=h\;du$, separates the in-boundary integral into the outer *u* integral and M(u). Including unavailable-link periods gives:

(23) $${\bar{P}}_{e}=\frac{1}{2}P_{\mathrm{out}}+\frac{B{\left(\alpha \beta \right)}^{(\alpha +\beta )/2}}{\Gamma (\alpha )\Gamma (\beta )}\int_{1/A_{0}}^{u_{\max}}u^{(\alpha +\beta )/2-1}\sum_{m=0}^{\infty }\sum_{n=0}^{\infty }\left(\Phi \left(m,n\right)g_{m,n}\left(1/u\right)M\left(u\right)\right)du$$

where

(24) $$M\left(u\right)=\int_{0}^{\infty }h^{(\alpha +\beta )/2-1}\mathrm{erfc}\left(h\sqrt{\frac{\mathrm{SNR}}{4}}\right)K_{\alpha -\beta }\left(2\sqrt{\alpha \beta uh}\right)dh$$

The inner turbulence integral M(u) has the following Meijer G-function representation, leaving one outer numerical integration:

(25) $$M\left(u\right)=\frac{2^{\alpha +\beta -3}}{\pi^{3/2}{\left(\alpha \beta u\right)}^{(\alpha +\beta )/2}}G_{5,2}^{2,4}\left[\frac{4\mathrm{SNR}}{{\left(\alpha \beta u\right)}^{2}}\;|\;\begin{array}{c} \frac{1-\alpha }{2},\frac{2-\alpha }{2},\frac{1-\beta }{2},\frac{2-\beta }{2},1\\ 0,\frac{1}{2} \end{array}\right]$$

Terms 0≤m≤M and 0≤n≤N are retained. The bound applies only to the in-boundary series; $P_{\mathrm{out}}/2$ is evaluated separately. The constants $C_{3}$ and $r_{3}<1$ are defined in Appendix B.

The truncation error upper bound E(M,N) after truncating the double series to *M* and *N* terms is shown in Appendix B to satisfy:

(26) $$E\left(M,N\right)\leq \frac{2C_{3}}{{\left(1-r_{3}\right)}^{2}}r_{3}^{\min(M,N)+1},\;0<r_{3}<1$$

Retaining indices 0≤m≤M and 0≤n≤N requires (M+1)(N+1) series terms at each outer integration node. A direct evaluation of the finite sum in $g_{m,n}$ involves m+1 terms, giving a series-evaluation cost proportional to (N+1)(M+1)(M+2)/2 per node, excluding special-function evaluation. Higher accuracy may therefore increase the computational burden, particularly as $r_{3}$ approaches unity. Given the computational cost of evaluating the truncated series, the BER expression is better suited to offline link-performance analysis and parameter selection than to direct execution in the real-time tracking loop. Precomputed lookup tables or interpolation may enable future online use, but their runtime and approximation accuracy should be carefully evaluated before deployment.

To validate the truncation orders numerically, Equation (23) was evaluated for M=N=32–8192 and several unequal (M,N) pairs. The experiments use the fitted pitch/yaw parameters in Table 6, a 100 m link, $\theta_{\max}=10^{\circ }$, HPHAs of $2^{\circ }$, $6.47^{\circ }$, $7^{\circ }$, $7.47^{\circ }$, and $15^{\circ }$, and reference aligned received SNRs of 10, 20, and 30 dB, giving 15 test cases. The reference SNR is defined at a $7^{\circ }$ HPHA with unit turbulence gain. An independent polar-density integral, without the Appell series, provides the numerical reference. Increasing the quadrature from 128 pointing nodes and 16×16 Gamma nodes to 256 and 24×24, respectively, changes the reference BER by less than $2\times 10^{-15}$ in these cases.

**Figure 5:**
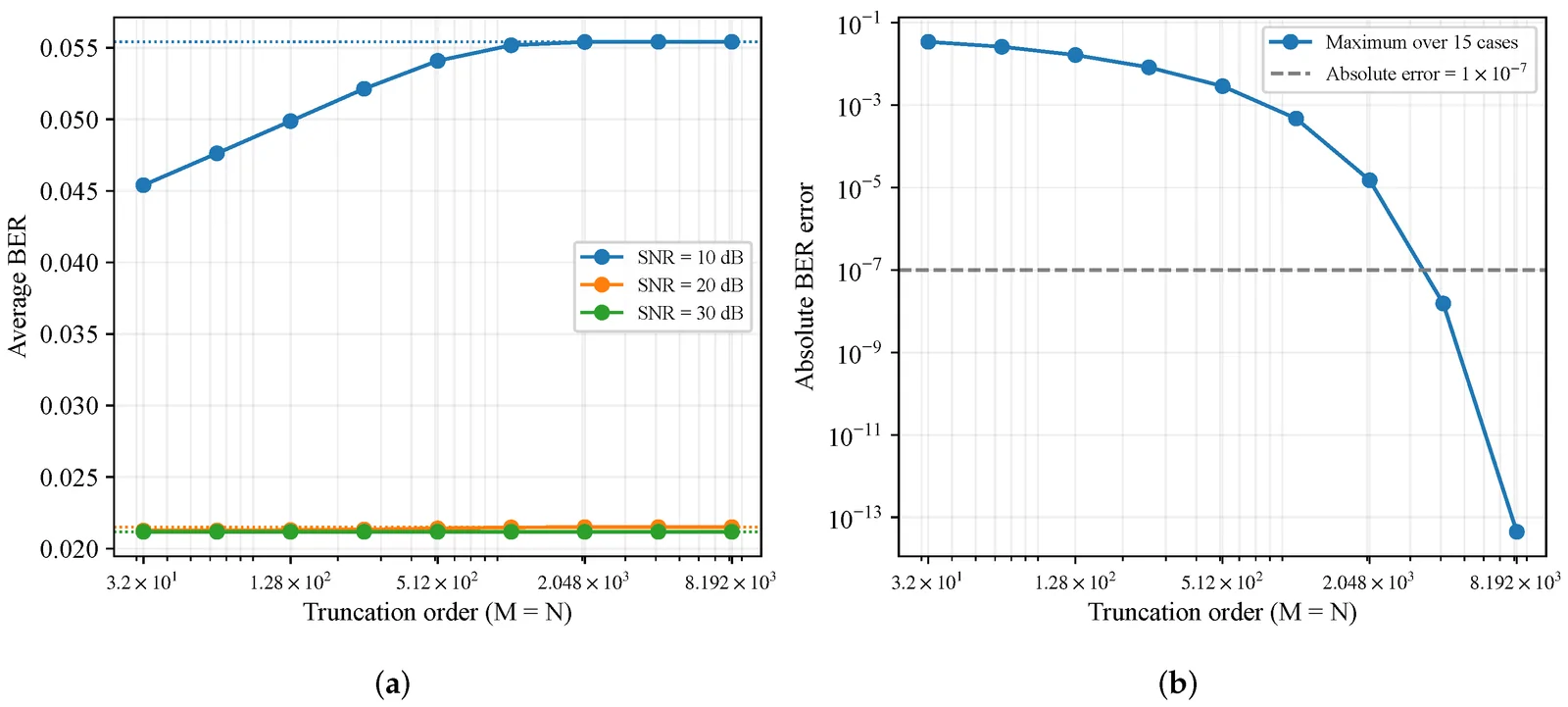
Numerical validation of Equation (23) truncation: (**a**) average BER versus M=N at a $7^{\circ }$ HPHA (dotted lines: independent numerical references), and (**b**) maximum absolute BER error across 15 HPHA–SNR combinations.

Figure 5 shows the BER convergence at a $7^{\circ }$ HPHA and the maximum absolute error over all 15 cases. For M=N=1024, 2048, and 4096, the maximum errors are approximately $4.74\times 10^{-4}$, $1.51\times 10^{-5}$, and $1.54\times 10^{-8}$, respectively; increasing both orders to 8192 reduces the error below $5\times 10^{-14}$. Unequal-order tests give errors of $2.61\times 10^{-2}$ for (M,N)=(64,4096), $1.21\times 10^{-6}$ for (M,N)=(4096,128), and $1.90\times 10^{-8}$ for (M,N)=(4096,256), demonstrating that the two orders need not have equal numerical requirements. The retained M=N=4096 configuration therefore meets an absolute BER tolerance of $10^{-7}$ in the tested scenarios. These numerical experiments complement the analytical bound and support offline computation.

## 3. Misalignment-Tolerant Transceiver and Link Maintenance Design

### 3.1. Transceiver Parameter Selection and Link Stability Boundaries

Designing fixed-wing inter-UAV OWC links requires balancing communication throughput, link stability, and platform SWaP constraints. The system must tolerate dynamic range variations, platform jitter, and atmospheric turbulence while keeping payload weight and power consumption minimal.

**Figure 6:**
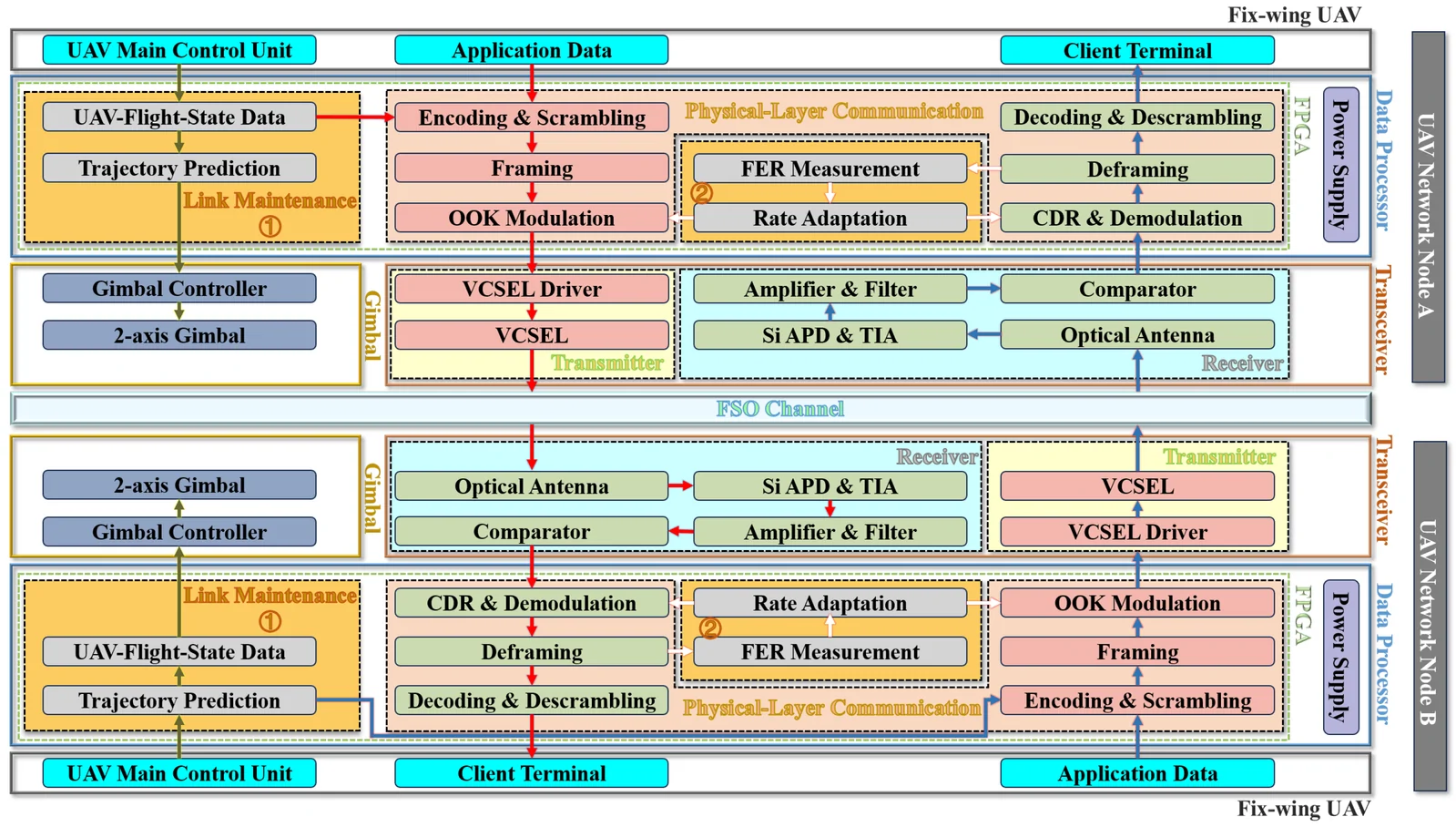
Schematic diagram of the proposed OWC system scheme for fixed-wing UAVs.

To maintain stable inter-UAV links under platform constraints and channel degradation, we propose a scheme based on a wide-angle NIR transceiver and a two-layer link maintenance strategy (Figure 6). The hardware includes the optical transceiver, a two-axis gimbal, and a communication processor. The receiver features a silicon avalanche photodiode (Si APD) and a transimpedance amplifier (TIA), while the transmitter is based on a 940-nm VCSEL. The communication processor utilizes a field-programmable gate array (FPGA) to implement both digital transceiver logic (framing, clock and data recovery (CDR), modulation/demodulation, Reed-Solomon (RS) coding) and tracking algorithms (telemetry integration, Kalman-filter-based feedforward pointing, and rate feedback protection). Combining the platform characteristics of the fixed-wing UAVs used in this work, Table 7 summarizes the inter-UAV OWC link parameters and corresponding system designs.

### 3.2. Airborne Integration of SWaP-Compliant Optical Transceiver

**Figure 7:**
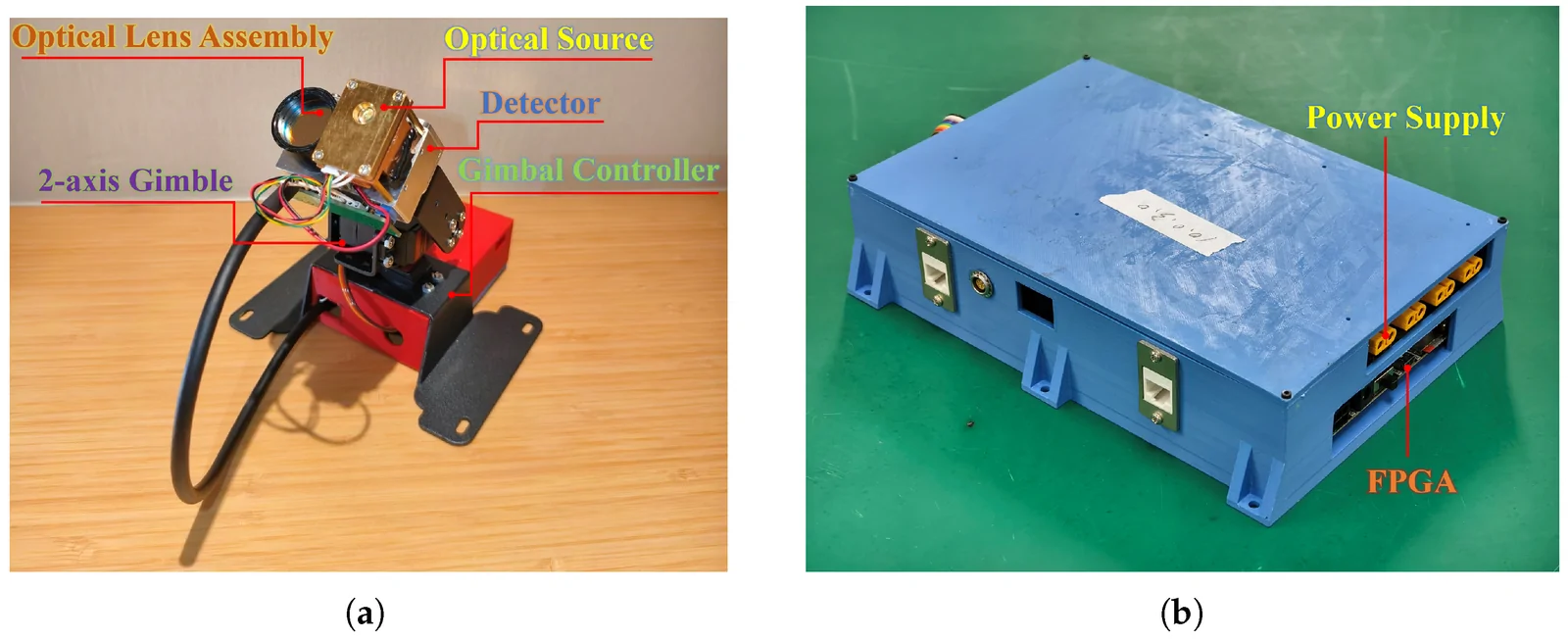
Airborne optical transceiver and signal processor: (**a**) optical transceiver structure, and (**b**) signal processing module.

To accommodate platform mobility, we utilize a NIR transceiver design featuring a divergent beam and a wide receiver field-of-view (FOV) to provide dual-sided pointing error tolerance. The standalone optoelectronic architecture, optical antenna design, and baseline link budget of the wide-angle NIR-OWC transceiver were established and experimentally validated in our prior work [26]. This intrinsic angular tolerance absorbs baseline platform attitude jitter, substantially relaxing active tracking requirements and enabling link maintenance with a lightweight two-axis gimbal. The transceivers are mounted on 120° coverage gimbals operating in the 940-nm near-infrared band. Key baseline parameters of the integrated transceiver (as characterized in [26]) are summarized in Table 8. Full-duplex operation at 940 nm is implemented using spatially separated transmit and receive apertures with approximately parallel optical axes, as illustrated in Figure 7a. The transmitter and receiver are mounted side by side with a longitudinal offset: the emitting aperture is positioned ahead of the receiving aperture and emits forward, preventing a direct optical path from the local transmitter into the local receiver. The two counter-propagating links therefore operate simultaneously through separate transmit and receive paths, with crosstalk suppression provided by the spatial arrangement rather than wavelength separation.

Although a divergent beam increases misalignment tolerance, it reduces receiver optical power density. To compensate for this geometric path attenuation, the transceiver incorporates the high-power transmitter module established in [26], which employs a 940-nm multi-emitter VCSEL array driven by a high-speed FET switching circuit in pulsed on-off keying non-return-to-zero (OOK-NRZ) modulation. A low-voltage differential signaling (LVDS) interface links the driver to the FPGA, where RS(255, 223) forward error correction is implemented to improve decoding sensitivity under intensity modulation with direct detection (IM/DD).

Laser safety of the underlying wide-divergence 940-nm transceiver was assessed in our previous work [26], which reported compliance with the IEC 60825-1:2014 [33] Class 1 requirements under the evaluated emission and encoding conditions. The present system retains the assessed optical configuration and 8b/10b encoding with a maximum continuous emission duration of 50 ns. The assessment accounts for beam divergence and accessible emission through the specified limiting apertures. We must nevertheless emphasize that, although the device meets the Class 1 requirements under the assessed conditions, prolonged direct viewing of the source and observation through optical instruments, such as microscopes or telescopes, should still be avoided.

At the receiver front-end, optical signals are collected by an objective lens, passed through a narrow optical bandpass filter (full-width at half-maximum ≈ 20 nm) to suppress solar background noise, and focused onto a silicon APD. The resulting photocurrent is amplified by a low-noise TIA and converted to digital LVDS signals, with a clock and data recovery (CDR) chip restoring digital timing before FPGA decoding.

The proposed system was developed with explicit consideration of platform SWaP constraints. The transceiver and controller each weigh 600 g, giving a combined mass of 1.2 kg, while the reported total system operating power is 70 W.

For the tested small fixed-wing UAV, which has a maximum payload capacity of 5 kg, this combined mass occupies 24% of the payload budget before accounting for additional installation hardware, leaving 3.8 kg of nominal capacity for mounting hardware and other equipment. The UAV carries four 30 Ah, 22.4 V battery packs, providing a total nominal energy of 2688 Wh. Taking the specified two-hour endurance as a baseline without the additional OWC load gives an estimated average flight power of 1344 W. Adding the 70 W system load reduces the estimated endurance to approximately 114 min, corresponding to a modest reduction of about 6 min or 5%. This simplified estimate considers electrical consumption only and excludes additional effects of equipment mass, installation drag, usable battery capacity, and power-conversion losses.

These estimates support the compatibility of the design with the tested platform and indicate remaining payload and power capacity. These figures suggest potential for adaptation within the mass and electrical-power budgets. Nevertheless, SWaP requirements become more restrictive on smaller fixed-wing platforms and may reduce the payload available for other instruments and the achievable endurance. Adaptation would require lighter mechanical packaging, more integrated electronics, and fewer optical terminals where network connectivity permits. Lower transmit power could further reduce the electrical and thermal burden, but would require corresponding trade-offs in communication range, data rate, or pointing tolerance.

The gimbal tracking system aligns the transceivers to maintain bidirectional links. The 2-axis gimbal provides elevation coverage of −30° to 90° and azimuth coverage of ±60° to cover different fields of view.

**Figure 8:**
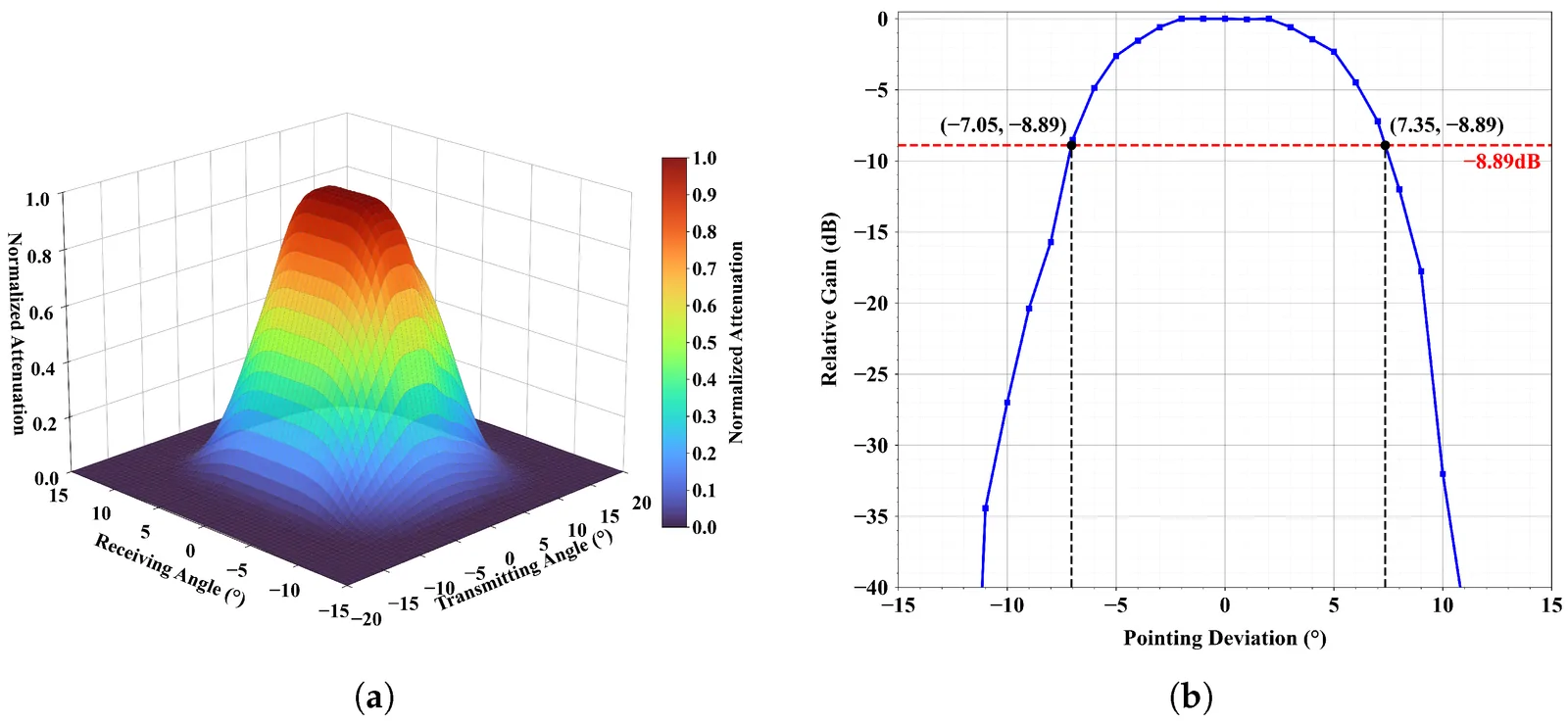
Pointing misalignment attenuation: (**a**) pointing attenuation versus transmitter divergence angle and receiver FOV, and (**b**) pointing error tolerance under 50 Mbps transmission rate and 100 m link range.

Figure 8a shows the pointing attenuation. At 50 Mbps and a 100 m range, the transceiver combination tolerates a combined pointing error of ±7.2° (Figure 8b). Based on the flight attitude dataset, the combined pointing error remains within this ±7.2° limit for 91.5% of the cruise phase. It should be noted that this 91.5% metric specifically represents the geometric angular pointing coverage provided by the wide-angle transceiver optics under platform attitude jitter, rather than the complete end-to-end link availability (which also depends on instantaneous atmospheric turbulence fading and path-loss margin). Nevertheless, this high angular coverage demonstrates that intrinsic optical tolerance alone accommodates the vast majority of cruise attitude jitter without relying on active gimbal tracking.

To further assess the system parameter selection, the design parameters are substituted into Equation (23) for numerical simulation. The centered pitch/yaw *t* parameters in Table 6 are used with independent axes. The link range, transmit on-symbol power, receiver diameter, and wavelength are 100 m, 4 W, 22 mm, and 940 nm, respectively; $C_{n}^{2}=1.7\times 10^{-14}$ m^−2/3^ is an assumed homogeneous near-ground scenario. Based on the measurements reported in [26] and the power margin estimated by inverse-square distance scaling, a pointing boundary of approximately 10° is adopted at 100 m. The contribution $P_{\mathrm{out}}/2$ is included without conditional normalization. Reference received SNR is defined for aligned pointing, unit turbulence gain, and a 7° HPHA; different SNR curves vary receiver noise while keeping transmit power fixed. The result is checked against independent polar-density quadrature; the maximum absolute BER difference is below $2\times 10^{-8}$ for this sweep. We select a daytime illuminance of 100,000 lux as a reference condition representing strong sunlight. With an assumed ground reflectance of 0.3 and a horizontal field of view divided equally between sky and ground, the scaled standard-spectrum estimate gives approximately 11.9 µW of filtered background power. With a 940-nm filter transmission of 96.74%, the aligned signal power at 100 m is approximately 2.50 µW/1.88 µW for the measured yaw/pitch HPHAs. Using the Hamamatsu S8664-30K silicon APD as a reference, with an assumed unmultiplied responsivity of 0.33 A/W and a 50 MHz equivalent noise bandwidth, the background-shot-noise-limited electrical SNR is approximately 31.5–34.0 dB for (M,x)=(50,0.2) and 28.9–31.4 dB for (100, 0.3), where $F=M^{x}$. Under the adopted OOK SNR convention, a conditional BER of 10^−3^ requires approximately 12.8 dB, yielding an aligned SNR margin of approximately 16.1–21.2 dB across these scenarios. It should be noted that this is a background-noise-limited design estimate, not a measured total receiver SNR or an average-link reliability guarantee; signal shot noise, dark current, and receiver-electronics noise are not included.

**Figure 9:**
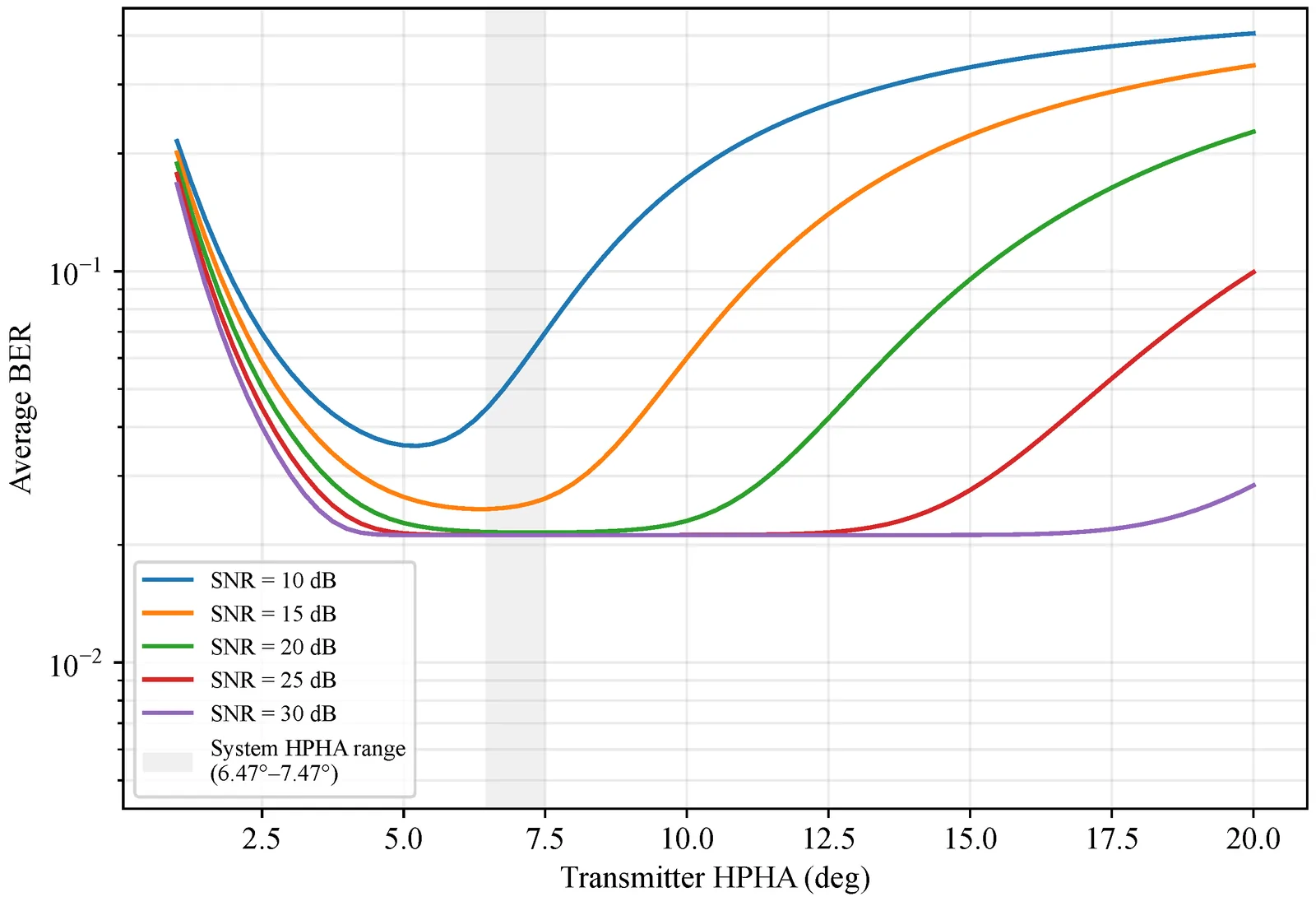
Average BER versus transmitter HPHA at 100 m, evaluated with $\theta_{\max}=10^{\circ }$ and an aligned receiver. SNR denotes reference aligned received SNR at a $7^{\circ }$ HPHA. The shaded span marks the measured yaw/pitch HPHAs.

Figure 9 evaluates the trade-off between pointing tolerance and geometric power dilution using Equation (23). In the evaluated 20–30 dB reference-SNR scenarios, the measured yaw/pitch HPHAs of 6.47°/7.47° give BER values within approximately 0.4% of the respective sweep minima. This supports the selected angular span as a practical passive-tolerance compromise within the integrated design. At higher SNR, the assumed communication-boundary contribution dominates and flattens the curves; low relative differences therefore do not establish superior performance. It should be noted that, for computational simplicity, the simulation assumes a circularly symmetric beam, with a 7° HPHA used as the SNR reference, whereas the actual beam has different yaw and pitch HPHAs. Residual pointing losses motivate the complementary pointing-compensation and rate-protection strategies.

To assess the scintillation level relevant to the flight trials, the H–V model is evaluated at heights of 130–140 m using reference values $C_{0}=1.7\times 10^{-14}$ m^−2/3^ and $v_{\mathrm{HV}}=21$ m/s. With a wavelength of 940 nm and an approximately homogeneous horizontal path, the resulting $C_{n}^{2}$ is (4.44–4.88) × 10^−15^ m^−2/3^. At 100 m, $\sigma_{R}^{2}$ = 0.00232–0.00256, α = 798.66–878.25, and β = 766.89–843.37; at 350 m, $\sigma_{R}^{2}$ = 0.02311–0.02541, α = 81.07–89.06, and β = 77.20–84.88. These values indicate weak turbulence under the assumed atmospheric profile. The normalized irradiance variance, SI=1/α+1/β+1/(αβ), ranges from approximately 0.0023 to 0.0254. The Gamma–Gamma model accommodates this weak-scintillation regime [31].

For an aligned reference received SNR of 30 dB, consistent with the order of magnitude of the preceding link-budget estimate, the scintillation contribution was assessed by comparing the average BER with Gamma–Gamma fading against that with $h_{a}=1$. Using a 7° HPHA, the fitted pitch/yaw pointing statistics, and the 10° communication boundary, the absolute BER increase over the evaluated 100–350 m and 130–140 m cases is below 10^−8^, corresponding to a relative increase below 0.001%. The comparison holds the aligned received SNR fixed to isolate scintillation. Thus, scintillation has a negligible effect on the overall average BER in these evaluated conditions, where the pointing-related unavailable-link contribution dominates.

Finally, deterministic atmospheric attenuation and solar background noise are considered separately. The reported trial-day visibility was approximately 50 km. Using the visibility-based extinction relation [34] and an assumed wavelength-scaling exponent of 1.3, the estimated extinction coefficient at 940 nm is $\kappa_{940}=\left[-\ln\left(0.02\right)/V\right]{\left(940/550\right)}^{-1.3}=0.0390$ ${\mathrm{km}}^{-1}$ for V=50 km. Beer–Lambert transmission then gives losses of approximately 0.017 dB at 100 m and 0.059 dB at 350 m. For comparison, the geometric loss for a 7° HPHA and a 22 mm aperture is approximately 62.5–73.4 dB over these ranges, while 5° and 10° pointing errors introduce approximately 1.5 and 6.1 dB of additional loss. The estimated clear-air extinction is therefore small relative to these losses and is neglected as an engineering approximation for the reported trials. This visibility-based estimate concerns aerosol-dominated extinction; molecular absorption was not independently measured and is not determined by visibility.

Solar background is treated as receiver noise rather than propagation loss. Under the standard-spectrum, filter-transmission, and viewing assumptions stated above, increasing illuminance from 10,000 to 100,000 lux raises the estimated filtered background power from approximately 1.19 µW to 11.88 µW. The APD background shot-noise variance is $\sigma_{i,\mathrm{bg}}^{2}=2qM^{2}FR_{0}P_{\mathrm{bg}}B$ [35], where *q* is the electron charge. With $R_{0}=0.33$ A/W, B=50 MHz, and the two gain/noise-index scenarios specified above, the equivalent input optical RMS noise is approximately 11–15 nW at 10,000 lux and 36–48 nW at 100,000 lux. Thus, solar background is not neglected: spectral filtering and restricted angular collection reduce it, and its shot noise is included in the link-budget estimate. These values include background shot noise only; total receiver noise also depends on signal shot noise, dark current, and the TIA. The 940-nm operating band is not solar-blind.

### 3.3. Two-Layer Link Maintenance and Gimbal Tracking Strategy

To enhance link stability, we propose a two-layer link maintenance scheme. The feedforward layer uses real-time GPS and attitude telemetry to predict relative motion and compensate for pointing errors in real time. The feedback protection layer dynamically adjusts the transmission rate based on the current data link quality to safeguard reliability under deep fades. We evaluate this strategy through Monte Carlo simulations based on real flight log data.

In the feedforward layer, telemetry from the flight controller is sent to the FPGA. A Kalman filter predicts the relative position and velocity between the local and target UAVs in the inertial frame. Let the relative state vector at time step *k* be defined as follows:

(27) $$x\left(k\right)=\left[\begin{array}{c} p(k)\\ v(k) \end{array}\right]={\left[\begin{array}{c} x\left(k\right),y\left(k\right),z\left(k\right),v_{x}\left(k\right),v_{y}\left(k\right),v_{z}\left(k\right) \end{array}\right]}^{T}$$

where $p\left(k\right)={\left[x\left(k\right),y\left(k\right),z\left(k\right)\right]}^{T}\in R^{3}$ denotes the three-dimensional relative position vector (along the North, East, and Up axes in the local Cartesian inertial frame), and $v\left(k\right)={\left[v_{x}\left(k\right),v_{y}\left(k\right),v_{z}\left(k\right)\right]}^{T}\in R^{3}$ denotes the corresponding relative velocity vector between the two UAVs at time step *k*. Based on the constant velocity (CV) motion model with a sampling interval of Δt, the state transition is x(k)=Fx(k−1)+w(k), and the observation model for the delayed telemetry is $z\left(M-1\right)=Hx\left(M-1\right)+n_{\mathrm{meas}}\left(M-1\right)$, where the observation matrix is $H=I_{6\times 6}$ and $n_{\mathrm{meas}}∼N\left(0,R\right)$ is the zero-mean Gaussian measurement noise vector (distinguished from the velocity vector v). The transition matrix F, measurement noise covariance R, and process noise covariance Q are defined as follows:

(28) $$F=\left[\begin{array}{cc} I_{3\times 3} & \Delta tI_{3\times 3}\\ 0_{3\times 3} & I_{3\times 3} \end{array}\right],\;R=\left[\begin{array}{cc} \sigma_{p}^{2}I_{3\times 3} & 0_{3\times 3}\\ 0_{3\times 3} & \sigma_{v}^{2}I_{3\times 3} \end{array}\right],\;Q=\left[\begin{array}{cc} \frac{\Delta t^{3}}{3}\sigma_{a}^{2}I_{3\times 3} & \frac{\Delta t^{2}}{2}\sigma_{a}^{2}I_{3\times 3}\\ \frac{\Delta t^{2}}{2}\sigma_{a}^{2}I_{3\times 3} & \Delta t\sigma_{a}^{2}I_{3\times 3} \end{array}\right]$$

The state estimate $\hat{x}$ is updated and predicted forward to time step *M* using standard discrete-time Kalman filtering equations, yielding the prediction:

(29) $$\hat{x}\left(M|M-1\right)=F\hat{x}\left(M-1|M-1\right)$$

Specifically, the predicted relative position vector $\hat{p}\left(M\right)={\left[\hat{x}\left(M|M-1\right),\hat{y}\left(M|M-1\right),\hat{z}\left(M|M-1\right)\right]}^{T}$ is extracted from the predicted state vector $\hat{x}\left(M|M-1\right)$. The corresponding ideal line-of-sight (LoS) unit vector in the ground inertial reference frame at time *M* is expressed as follows:

(30) $$u\left(M\right)=\frac{\hat{p}\left(M\right)}{∥\hat{p}\left(M\right)∥}$$

This LoS unit vector is projected into the aircraft body frame using the real-time attitude angles $\eta \left(M\right)={\left(\theta \left(M\right),\phi \left(M\right),\kappa \left(M\right)\right)}^{T}$ measured by the onboard sensors:

(31) $$u^{b}\left(M\right)=R_{G2b}\left(\eta \left(M\right)\right)u\left(M\right)={\left[\begin{array}{ccc} u_{x}^{b}\left(M\right) & u_{y}^{b}\left(M\right) & u_{z}^{b}\left(M\right) \end{array}\right]}^{T}$$

where $R_{G2b}\left(\eta \left(M\right)\right)$ is the coordinate transformation matrix from the ground inertial reference frame to the aircraft body coordinate system. Based on the projected LoS vector, the target gimbal azimuth $\alpha^{*}\left(M\right)$ and pitch $\beta^{*}\left(M\right)$ are derived as follows:

(32) $$\alpha^{*}\left(M\right)=\mathrm{atan}2\left(u_{y}^{b}\left(M\right),u_{x}^{b}\left(M\right)\right),\;\beta^{*}\left(M\right)=-\arcsin\left(u_{z}^{b}\left(M\right)\right)$$

The relative LoS vector change between time steps M−1 and *M* is:

(33) $$\Delta u^{b}=R_{G2b}\left(\eta \left(M\right)\right)u\left(M\right)-R_{G2b}\left(\eta \left(M-1\right)\right)u\left(M-1\right)$$

This difference is decomposed into platform rotation ($u_{\mathrm{att}}^{b}$) and relative translation ($u_{\mathrm{traj}}^{b}$) components:

(34) $$\Delta u^{b}=u_{\mathrm{att}}^{b}+u_{\mathrm{traj}}^{b}$$

where $u_{\mathrm{att}}^{b}=\left[R_{G2b}\left(\eta \left(M\right)\right)-R_{G2b}\left(\eta \left(M-1\right)\right)\right]u\left(M-1\right)$ represents the LoS change induced solely by the platform attitude variation, and $u_{\mathrm{traj}}^{b}=R_{G2b}\left(\eta \left(M\right)\right)\left[u(M)-u(M-1)\right]$ represents the change caused by the relative translational trajectory. Using a first-order Taylor expansion to linearize the mapping from body-frame coordinates to gimbal angles, the tracking error relation is expressed in transpose form as follows:

(35) $${\left[\begin{array}{cc} \Delta \alpha & \Delta \beta \end{array}\right]}^{T}\approx J\left(\alpha ,\beta \right){\left[\begin{array}{ccc} \Delta u_{x}^{b} & \Delta u_{y}^{b} & \Delta u_{z}^{b} \end{array}\right]}^{T}$$

where the Jacobian matrix J(α,β) is defined as follows:

(36) $$J\left(\alpha ,\beta \right)=\left[\begin{array}{ccc} -\frac{\sin\alpha }{\cos\beta } & \frac{\cos\alpha }{\cos\beta } & 0\\ 0 & 0 & -\frac{1}{\cos\beta } \end{array}\right]$$

The final control command increment at time *M* is:

(37) $${\left[\begin{array}{cc} \Delta \alpha (M) & \Delta \beta (M) \end{array}\right]}^{T}=J\left(\alpha \left(M-1\right),\beta \left(M-1\right)\right)\left(u_{\mathrm{att}}^{b}+u_{\mathrm{traj}}^{b}\right)+e\left(M-1\right)$$

where $e\left(M-1\right)={\left[\alpha^{*}\left(M-1\right)-\alpha \left(M-1\right),\beta^{*}\left(M-1\right)-\beta \left(M-1\right)\right]}^{T}$ is the tracking error vector of the 2D gimbal.

**Figure 10:**
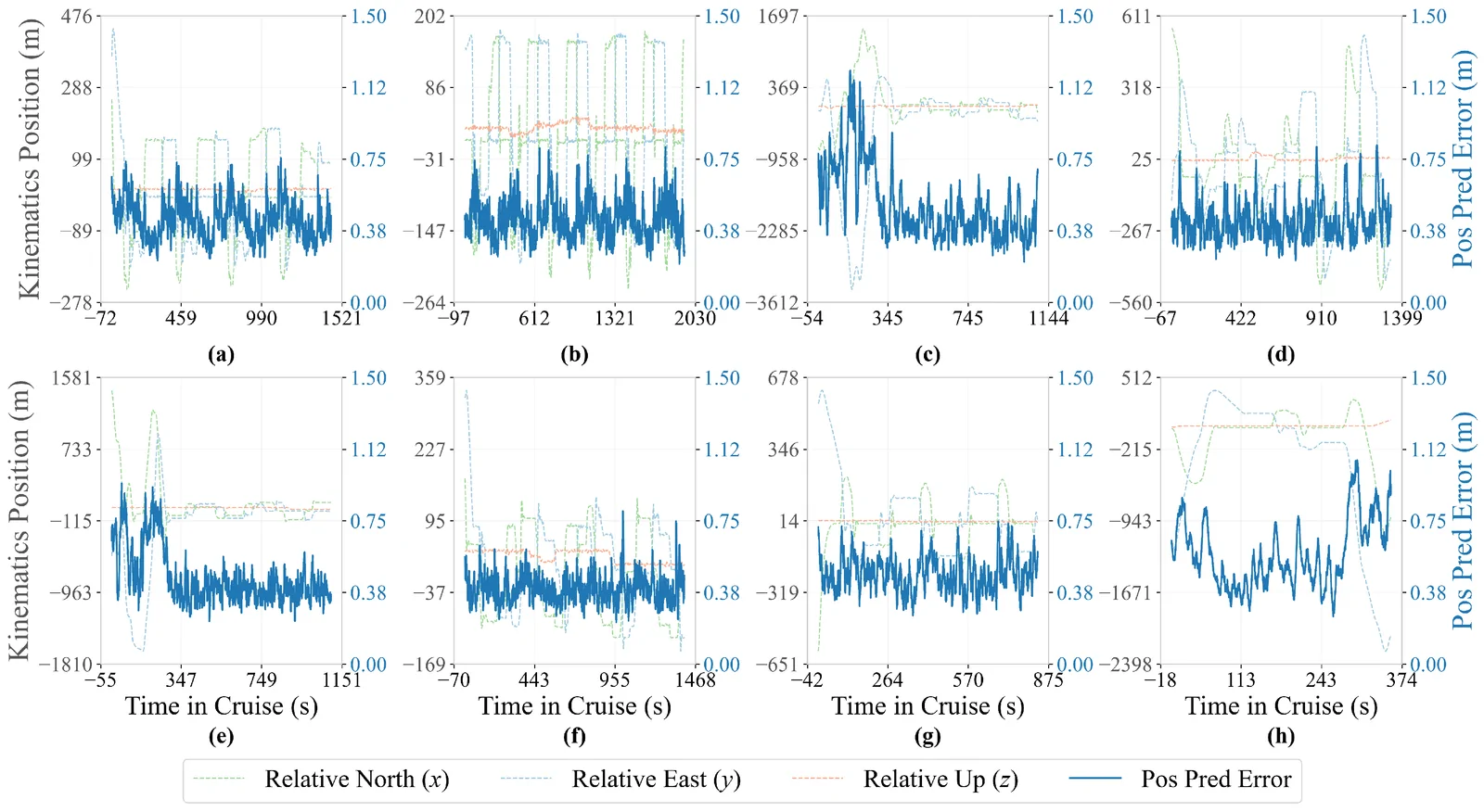
Relative position estimation error under Kalman filtering across the eight airborne communication flight trials: (**a**) flight record UART_079 on 14 January 2025; (**b**) UART_081 on 14 January 2025; (**c**) UART_012 on 15 January 2025; (**d**) UART_014 on 15 January 2025; (**e**) UART_091 on 15 January 2025; (**f**) UART_094 on 15 January 2025; (**g**) UART_071 on 18 January 2025; and (**h**) UART_075 on 18 January 2025.

**Figure 11:**
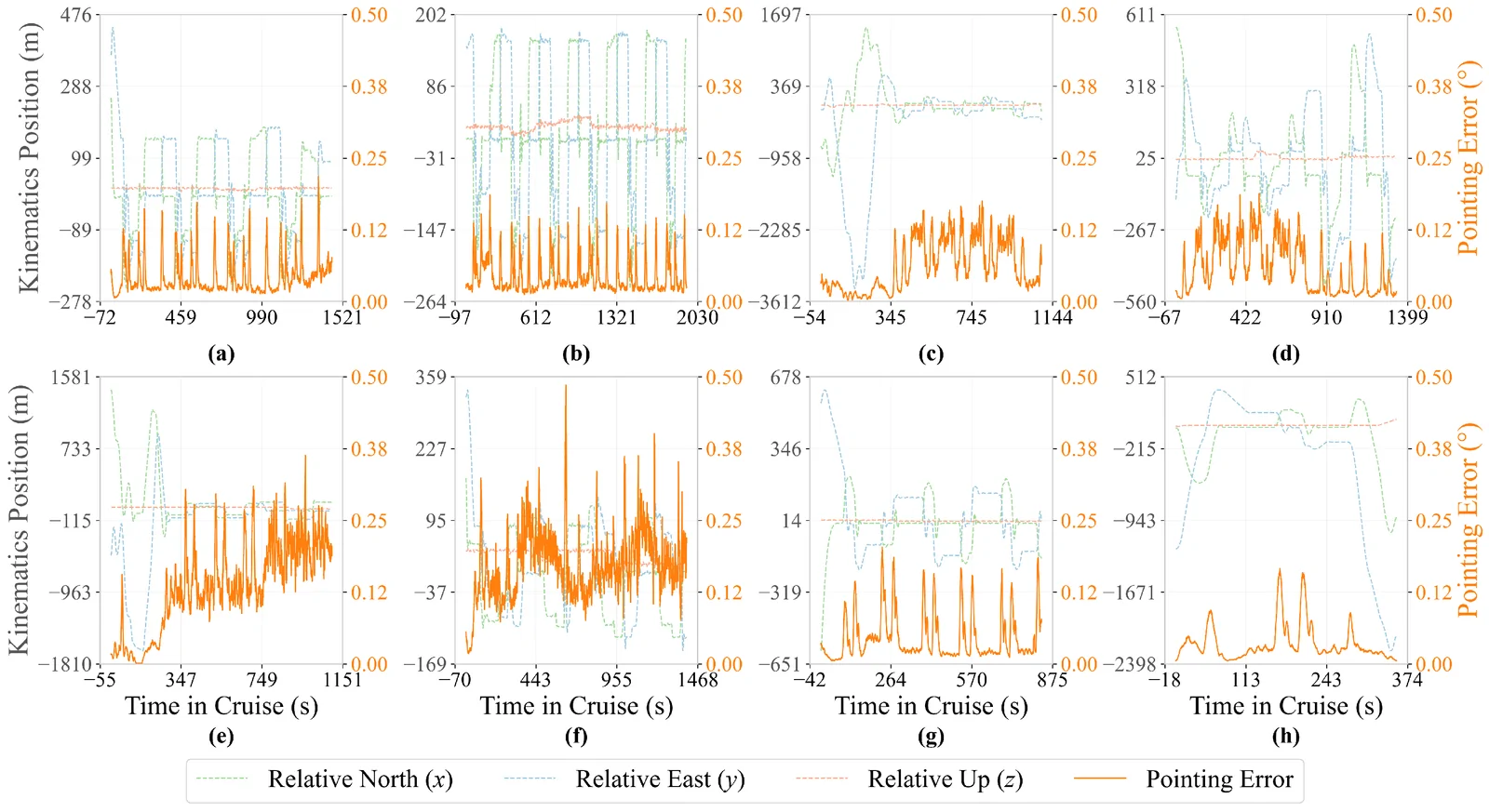
Target pointing error across the eight airborne communication flight trials: (**a**) flight record UART_079 on 14 January 2025; (**b**) UART_081 on 14 January 2025; (**c**) UART_012 on 15 January 2025; (**d**) UART_014 on 15 January 2025; (**e**) UART_091 on 15 January 2025; (**f**) UART_094 on 15 January 2025; (**g**) UART_071 on 18 January 2025; and (**h**) UART_075 on 18 January 2025.

Furthermore, Monte Carlo simulations verify the algorithm’s effectiveness. While Section 2.2 analyzed the full 15-flight attitude jitter dataset across all available sorties (comprising single-UAV and formation flights totaling 697.2 min flight endurance and 237.1 min of steady straight cruise), the dual-UAV relative positioning and pointing tracking simulation in this section specifically requires paired formation flight records containing synchronized leader–follower telemetry. Among the 15 flight logs, exactly eight sorties correspond to follower aircraft executing formation tracking and logging synchronized real-time leader kinematics; the remaining seven sorties represent single-UAV flights or leader aircraft that do not track an external target. These eight follower datasets were chosen based on their substantial flight endurance (total flight time ranging from 39.5 to 54.8 min, with an average of 48.4 min), extended cruise duration (7.4 to 40.3 min, with an average of 24.7 min), and prolonged steady straight cruise tracking intervals (stable straight-line cruise durations ranging from 3.9 to 13.4 min, with an average of 10.6 min). Raw telemetry recorded at 50 Hz is downsampled to 10 Hz (dt=0.1 s) to conform to the discrete inter-UAV telemetry link update rate. Cruise flight intervals (ground speed >15 m/s) are extracted, and the initial and terminal 10% transition phases are trimmed to isolate the middle 80% steady cruise phase (lasting 5.9 to 32.2 min per flight) from boundary climb/descent transients. To evaluate sustained tracking performance and filter high-frequency discrete sample measurement noise, a 5-s (50-sample) moving average is applied to the time series. Configuration parameters are summarized in Table 9, and the resulting error curves are shown in Figure 10 and Figure 11.

Simulation results show that relative position estimation and target pointing remain stable. Both errors are strongly correlated with the flight heading, exhibiting periodic fluctuations and transient spikes during maneuvers that rapidly decay once the UAV enters straight cruise. Across the eight simulated flight trials, the 5-s moving-average filtered relative position estimation root-mean-square (RMS) error is 0.466 m (mean 0.449 m, standard deviation 0.125 m, maximum 1.214 m), and the target pointing RMS error is 0.103° (mean 0.077°, standard deviation 0.068°, maximum 0.485°), strictly matching the response curves and scale ranges depicted in Figure 10 and Figure 11. For the raw discrete 10 Hz telemetry samples prior to moving-average filtering, the pointing RMS error is 0.125° (mean 0.078°, standard deviation 0.097°, and maximum instantaneous spike 1.810°). To ensure a statistically consistent comparison between pre- and post-compensation performance, the baseline uncompensated pointing misalignment is defined by the composite root-sum-square (RSS) standard deviation of the natural 3D platform attitude jitter, $\sigma_{\mathrm{comp}}=\sqrt{\sigma_{p}^{2}+\sigma_{y}^{2}+\sigma_{r}^{2}}\approx 6.08^{\circ }$ (which also equals an RMS jitter of 6.08° under the zero-mean condition μ≈0). Comparing identical statistical metrics, the feedforward compensation suppresses the pointing RMS error from the uncompensated baseline of 6.08° to 0.103° (0.125° for raw discrete samples), and reduces the error standard deviation from 6.08° to 0.068° (0.097° for raw discrete samples). This represents an error suppression of over 98%, successfully preventing link outage during flight maneuvers.

The rate-adaptive feedback layer monitors the frame error rate (FER) to dynamically scale the communication rate. When the FER exceeds a scale-down threshold, the rate is reduced, decreasing the receiver SNR requirement to maintain basic telemetry and data transmission under severe fading. Conversely, if the FER remains low, the rate is scaled up. This ensures the delivery of critical flight telemetry needed for gimbal alignment even during extreme channel degradation, preserving feedforward tracking loop integrity.

## 4. Experimental Platform and Airborne Flight Trial Verification

### 4.1. Experimental Platform and Scenario Setup

**Figure 12:**
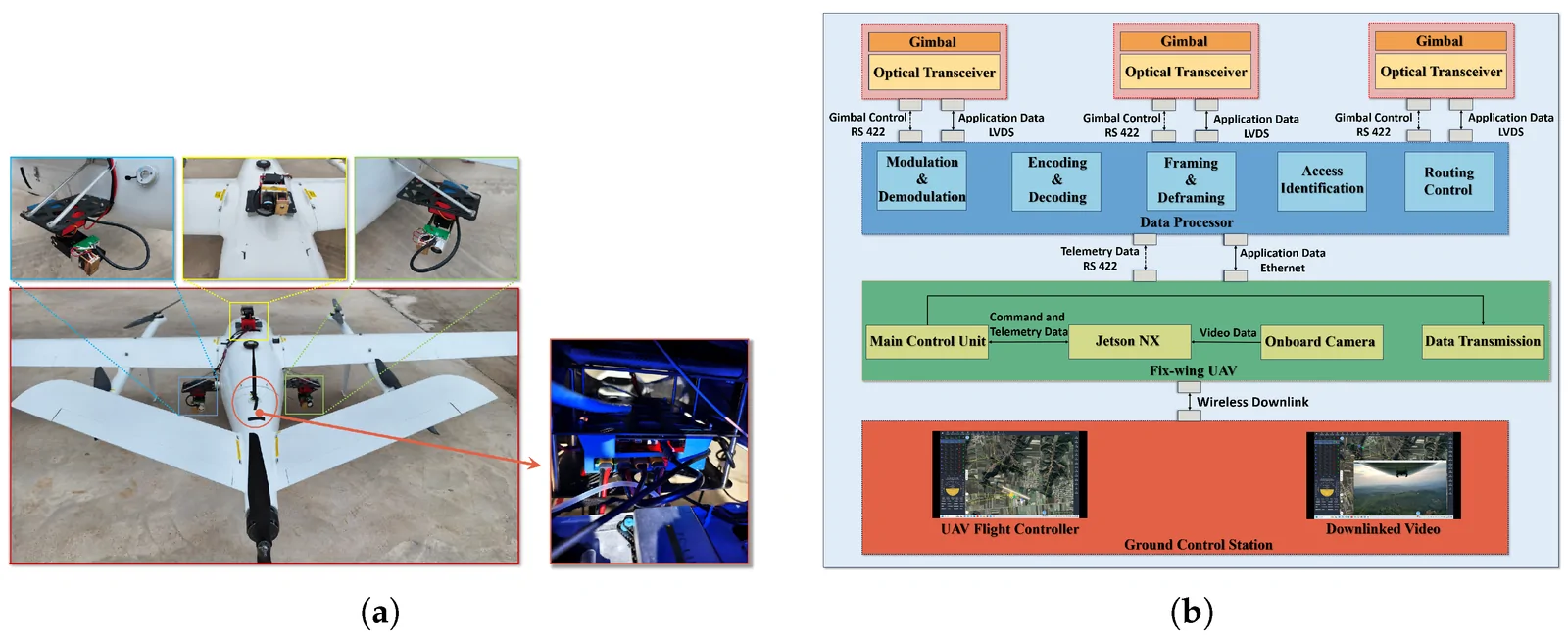
Airborne demonstration verification node: (**a**) installation of the optical transceiver on the UAV, and (**b**) hardware signal data flow.

The experimental verification system was constructed using three modified INNNO ING-3500 fixed-wing UAV platforms, each serving as an airborne communication node. Each node was equipped with an OWC prototype consisting of a central signal processing module and three optical transceiver terminals. Each terminal integrates a near-infrared transceiver and a two-axis gimbal. The payload of each UAV also included a flight control system, a standard RF data link, an onboard gimbal camera (acting as the business data source), and an NVIDIA Jetson Xavier NX processor. To cover wide spatial fields of view, the three transceivers on each aircraft were mounted on the fuselage back and the two wingtips, respectively (Figure 12a). All transceivers were connected to the OWC controller to facilitate multi-directional data transmission, reception, and dynamic routing.

The detailed node architecture and data flow within the network operate as follows (Figure 12b): business data is generated by the source node’s gimbal camera, processed by the Jetson NX, and transmitted via Ethernet to the local OWC signal processing module. The module performs framing, encoding, and modulation, outputting the data as an LVDS stream to the designated transceiver according to the routing decision, which then transmits the optical signal. At an intermediate relay node, the incoming optical signal is converted to an LVDS signal by the receiver and forwarded to the local processor. The processor demodulates and decodes the stream to parse the routing header and determine its destination. If the packet is destined for another node, it is re-encoded, re-modulated, and routed to the optical transceiver oriented toward the target direction. Upon reaching the destination node, the receiver converts the optical signal back to LVDS for decoding and deframing by the local processor. Once the local node is verified as the target destination, the business data is repackaged and routed via Ethernet to the Jetson NX, which downlinks the data to the ground station via the RF telemetry link.

It should be noted that, due to telemetry integration limitations of the UAV autopilot during the trials, real-time flight telemetry could not be directly parsed by the OWC signal processor. Therefore, the feedforward sensing layer proposed in Section 3.3 was not implemented in the current flight trials. Telemetry coupling and active feedforward tracking remain subjects of ongoing development. The flight trials were conducted at a designated airfield between 9:00 and 17:00 under varied solar illuminance (10,000–100,000 lux) and ambient temperatures ranging from −10 °C to 40 °C. The key operational parameters of the system are summarized in Table 10.

Initially, two modified UAV platforms, designated as UAV 1 and UAV 2, were deployed to validate OWC performance in dynamic flight states. UAV 1 served as the source node carrying the gimbal camera, while UAV 2 acted as the destination node equipped with the RF downlink. Video data generated by UAV 1 was transmitted across the OWC link to UAV 2, which then forwarded the stream to the ground station via its RF link.

Prior to flight, ground integration and simulated formation tests were conducted to verify system functionality. The two UAVs were placed in a tandem configuration on the runway to simulate their spatial relationship in level flight. The right-wing gimbal of UAV 1 pointed backward, and the fuselage-top gimbal of UAV 2 pointed forward. In our prototype architecture, data framing continuously operates at the preset system communication rate. The transmitted data stream is composed of business payload and pre-set dummy frames, within which periodic routing and handshake control frames are multiplexed. When no business payload is present, dummy and routing frames occupy the entire transmission bandwidth. When business data is generated, business frames are assigned strict transmission priority, while the remaining bandwidth is continuously padded with dummy frames. Consequently, both the network aggregate throughput and the physical link transmission rate are consistently maintained at the preset rate (the same principle applies to other operational system communication rates). Furthermore, the network routing and link control mechanism strictly requires a continuous bidirectional handshake between the two nodes to establish and sustain data transmission.

**Figure 13:**
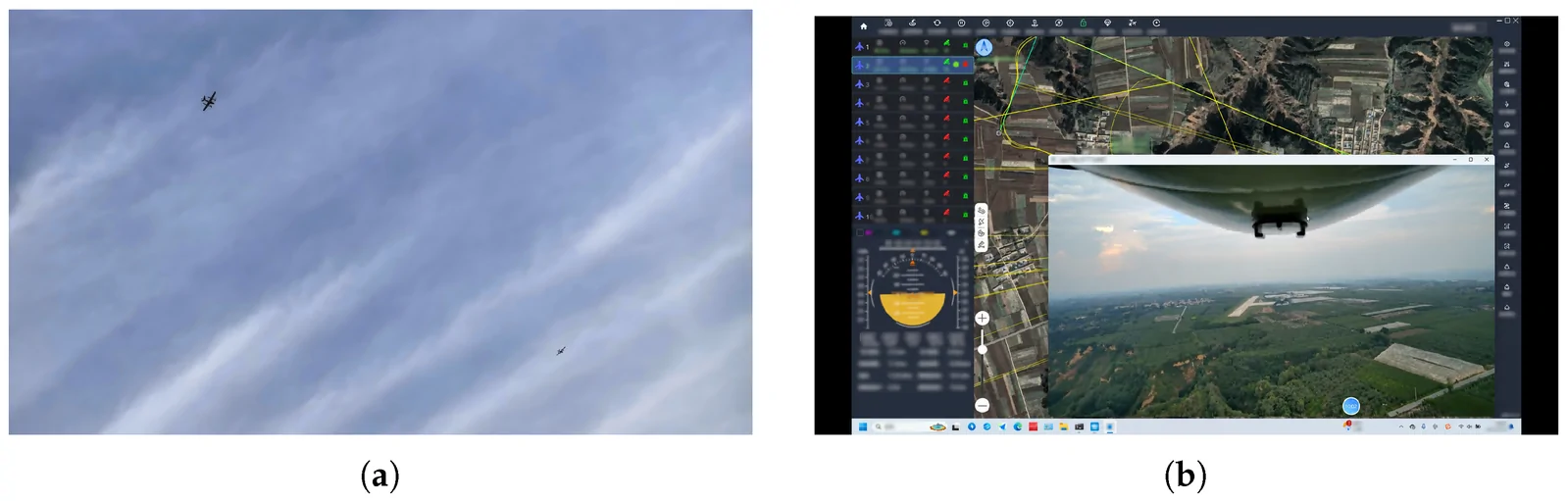
Two-UAV airborne communication flight trial and real-time video transmission: (**a**) two-UAV longitudinal line formation, and (**b**) downlink video feed received at the ground station.

During the flight experiment, the two aircraft took off sequentially and established a leader-follower formation, maintaining a longitudinal separation of approximately 100 m and a vertical offset of 10 m (Figure 13). Constrained by the directional coverage of the ground station antenna, steady straight flight segments were limited to approximately 2 km per run, corresponding to a single-pass cruise duration of approximately 1 min.

To evaluate multi-node relaying and network performance, we subsequently designed a three-UAV triangular formation.

**Figure 14:**
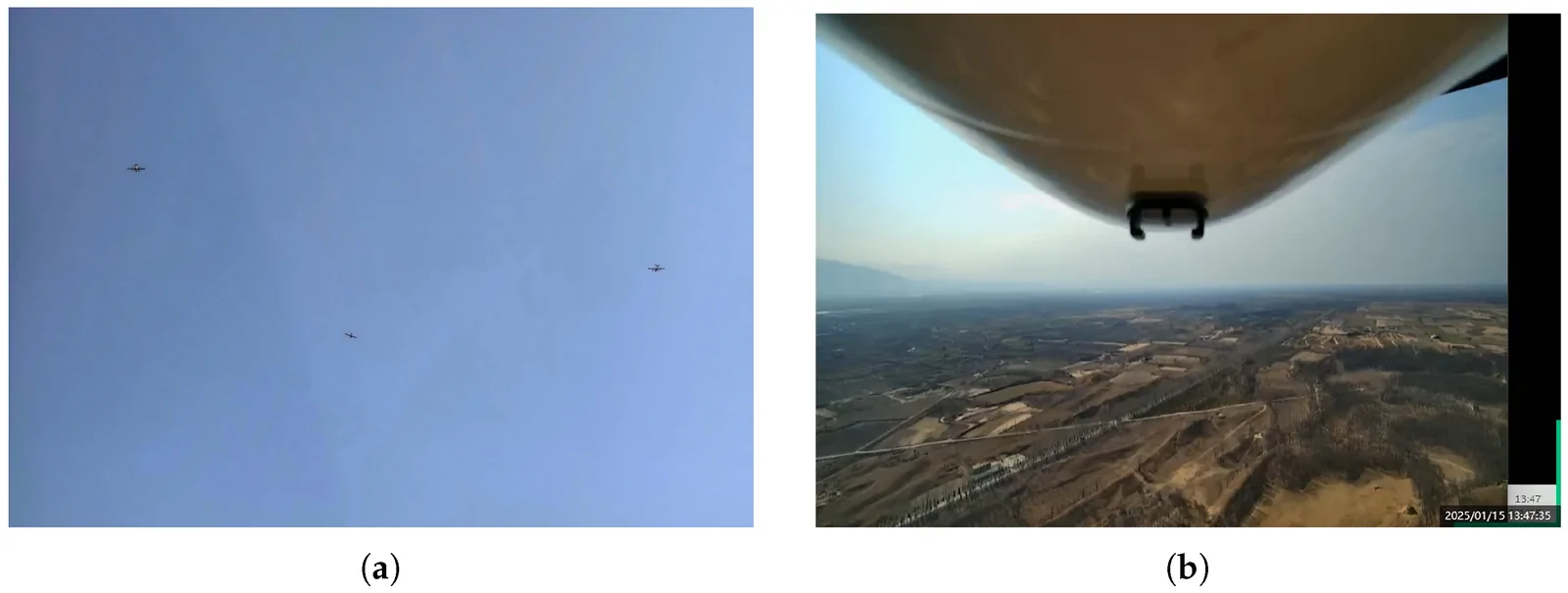
Three-UAV airborne communication flight trial and real-time video transmission: (**a**) three-UAV triangular formation, and (**b**) downlink video feed received at the ground station.

During the flight trials, UAV 1 cruised as the leader at an altitude of 140 m. Using a local coordinate frame centered on UAV 1 (where the flight direction defines the *X*-axis, left-lateral perpendicular to the fuselage defines the *Y*-axis, and the upward normal defines the *Z*-axis), the target formation coordinates were set to (0,120,−40) m for UAV 2 and (−300,−50,−30) m for UAV 3. The aircraft established the triangular formation sequentially (Figure 14). It should be clarified that the figures 120 m and 300 m represent nominal target coordinate offsets along the lateral (*Y*) and longitudinal (−X) axes, respectively, rather than direct inter-node link distances.

It should be noted that this rate reduction was performed manually by the operators via ground telecommand rather than automatically by an online closed-loop feedback algorithm, as real-time FER-driven dynamic rate switching had not yet been integrated into the onboard flight control loop.

The autopilot used an independent sequential tracking strategy, requiring each follower to wait for its leader to stabilize before initiating tracking.

### 4.2. Airborne Communication Flight Trial Results and Analysis

The successful transmission of the business payload across the optical link inherently confirms that the reverse optical link was concurrently active in maintaining the real-time bidirectional handshake. All data categories (business payload, dummy padding, and routing handshake frames) were thus simultaneously communicated in full duplex at the full system communication rate in both directions.

Across these straight cruise segments, the optical link maintained continuous connectivity in simultaneous full duplex, and real-time video transmission was observed at the ground station with smooth playback and negligible visual dropouts, as shown in the representative continuous recording of over 40 s in Visualization S1. This observation supports the feasibility of real-time video transmission during steady cruise.

Ground integration tests verified OWC relaying at 50 Mbps, where business video data from UAV 1 was successfully routed through UAV 2 (acting as the relay node) to UAV 3, and downlinked via the wireless data link of UAV 3.

In the relay topology where data is routed from UAV 1 through UAV 2 to UAV 3, the actual three-dimensional (3D) Euclidean distance for the first hop (UAV 1 to UAV 2) is calculated as $d_{12}=\sqrt{0^{2}+120^{2}+{\left(-40\right)}^{2}}\approx 126.5$ m (exceeding 120 m). For the second hop (UAV 2 to UAV 3), the relative inter-node displacement vector is (−300,−170,10) m, corresponding to an actual link distance calculated as $d_{23}=\sqrt{{\left(-300\right)}^{2}+{\left(-170\right)}^{2}+10^{2}}\approx 345.0$ m in 3D Euclidean distance (exceeding 300 m). This results in a cumulative two-hop optical path length of $d_{12}+d_{23}\approx 471.5$ m.

At the initial operational system communication rate of 50 Mbps, the real-time video stream received at the ground station exhibited noticeable packet dropouts, frame freezes, and screen tearing during transient maneuvers. Continuous and stable video transmission was achieved and approached the boundary of stable communication once the system communication rate was switched to 20 Mbps (Visualization S2, showing a representative segment of stabilized playback). At the 20 Mbps system communication rate, the video payload (exceeding 8 Mbps) accounted for over 40% of the channel throughput (with the remaining bandwidth padded by dummy and routing handshake frames). Consequently, this result serves as an empirical experimental observation supporting the concept of rate adaptation: scaling down the transmission rate reduces the required receiver SNR and expands the link margin, confirming the fundamental feasibility of adaptive rate scaling under degraded channel states. This performance degradation at 50 Mbps is mainly attributed to two factors. First, constrained by the range of the ground station antenna, straight segments were limited to 2 km (lasting approximately 1 min), which did not allow all three UAVs to achieve simultaneous steady-state cruising. Consequently, the formation remained in a transient state characterized by severe attitude fluctuations and rapid topological changes, complicating link maintenance. Second, multi-hop relaying requires simultaneous connection across two independent optical links, which cascades the link failure probabilities.

In summary, the experimental results demonstrate that the proposed fixed-wing UAV OWC system successfully establishes bidirectional optical links and multi-hop relaying. The flight trials demonstrate stable data transmission during steady straight flight, supporting the ability of the misalignment-tolerant transceiver to accommodate platform jitter. Furthermore, the successful stabilization of video transmission upon manually switching the rate from 50 Mbps to 20 Mbps provides experimental evidence supporting the feasibility and concept of rate-adaptive protection. However, in complex dynamic maneuvers and multi-node networking, active link maintenance strategies, such as closed-loop telemetry-based feedforward tracking and automated rate adaptation, are crucial to ensure link robustness.

## 5. Conclusions

This study investigated inter-UAV OWC on small fixed-wing platforms through flight-jitter characterization, system integration, simulations, and flight trials. The main findings are summarized as follows:

(1) A public attitude dataset covering 15 flight sorties was established. The measured jitter was characterized using location-scale *t*-distributions and incorporated into a semi-analytical BER model accounting for pointing errors and Gamma-Gamma turbulence.
(2) A wide-angle transceiver was integrated with a two-layer link maintenance design. Flight-log-driven simulations yielded a feedforward pointing RMS error of $0.103^{\circ }$, supporting the feasibility of telemetry-based pointing compensation.
(3) Flight trials demonstrated full-duplex video transmission at 50 Mbps over a link distance exceeding 100 m during fixed-wing cruise, and three-UAV relaying at 20 Mbps over a cumulative range exceeding 300 m. The relay rate was adjusted manually; real-time feedforward tracking and automated rate adaptation were not enabled during these trials.

Taken together, the present study has several limitations: feedforward pointing and automated rate adaptation were not evaluated in real-time flight operation, and the flight trials did not yield valid records of received optical power, SNR, FER/BER, or time-resolved link availability. Moreover, the computational cost of the BER expression limits its direct online use. Future work will address these limitations by integrating onboard link-quality logging and telemetry downlink, implementing and flight-testing the two-layer link maintenance strategy, and assessing model robustness to extreme pointing events. Efficient numerical approximations will also be investigated for potential online use, alongside further trials involving maneuvers and dynamic multi-UAV networking.

## Figures and Tables

**Table 1:** Comparison of representative UAV OWC experimental demonstrations.

Ref.	Light Source	Rate	Range	Rate–Range (Mbps·m)	Scenario	SWaP	Tracking Strategy
[20]	UV LEDs on UAV arms	60 bps	17 m	0.00102	Rotary-wing, hovering and low-speed mobility	Communication-terminal mass, size, and power not reported	Distributed UV markers, two wide-FOV cameras, and Kalman-filter tracking
[21]	White LED	2 Mbps	13.1 m	26.2	Rotary-wing, nighttime and low-speed mobility	Communication module: 975 g; overall size and total power not reported	3-axis stable gimbal and visual tracking module
[22]	Fiber-fed 660-nm LD	2 Gbps	2 m	4000	Rotary-wing, daytime and indoor hovering	Communication-terminal mass, size, and power not reported	Manual calibration and collimated optical path
[23]	265-nm HTP LED array (4 LEDs/side)	9.6 kbps	6 m	0.0576	Rotary-wing, daytime and outdoor hovering	Tx/Rx assembly: each <1.5 kg; overall size and total power not reported	Hexagonal truncated pyramid (HTP) LED array and ACPC antenna
[18]	LD/EAM-MRR	500 Mbps	560 m	280,000	UAV–ground retroreflective link	Not reported	External illumination and onboard EAM-MRR
[24]	275-nm LED array	20 Mbps (test signal)	30 m (flight)	600 (test signal)	UAV–ground vehicle; daylight mobile video	64 W; mass and size not reported	Visual tracking and three-axis gimbal
[25]	DUV LED array	40 Mbps	10 m altitude; 100 m fixed link	4000 (fixed link)	UAV–ground broadcast; separate nighttime fixed-platform full-duplex test	1.82 kg; 220×140×100 mm; 32 W	Vision-based tracking and three-axis gimbal
This work	VCSEL	50 Mbps	100 m	5000	Fixed-wing, daytime and outdoor high-speed mobility	OWC Components: 600 g, 160×140×100 mm; Data processor: 600 g, 230×170×70 mm	Dual-sided misalignment tolerant transceiver and two-axis gimbal

**Table 2:** Key specifications of the INNNO ING-3500.

Parameter	Specification
Flight mode	VTOL
Power source	Pure electric
Wingspan × Length × Height	3.5 m × 1.5 m × 0.5 m
Takeoff weight	15.5 kg
Battery	30,000 mAh LiPo × 4
Max payload	5 kg
Max speed	120 km/h
Max endurance	2 h

**Table 3:** Summary of flight parameters in the attitude jitter dataset.

Parameter	Minimum	Maximum	Mean
Altitude (m)	87.15	165.65	131.51
Ground speed (m/s)	15.57	36.59	25.18
Airspeed (m/s)	6.33	33.46	25.82
Wind speed (m/s)	0.00	11.10	3.56
Single flight range (km)	12.54	64.06	39.81
Single flight duration (min)	33.86	54.79	46.48

**Table 4:** Correlation between 3D attitude jitter and flight parameters.

Parameter	Pitch Std Dev	Yaw Std Dev	Roll Std Dev
Mean Airspeed	r=−0.232, p=0.404	r=−0.010, p=0.972	r=0.142, p=0.614
Airspeed Std Dev	r=0.521, p=0.047	r=0.148, p=0.598	r=0.390, p=0.150
Mean Acceleration	r=−0.568, p=0.027	r=−0.012, p=0.965	r=−0.261, p=0.348
Mean Wind Speed	r=−0.271, p=0.328	r=0.647, p=0.009	r=0.535, p=0.040
Wind Speed Std Dev	r=0.123, p=0.662	r=0.602, p=0.017	r=0.425, p=0.114

**Table 5:** Goodness-of-fit test results of attitude angle distributions.

Distribution	KS Statistic			KL Divergence		
	Pitch	Yaw	Roll	Pitch	Yaw	Roll
Normal	0.0510	0.2282	0.1781	0.0380	0.6596	0.4295
Laplace	0.0175	0.0815	0.0664	0.0078	0.1975	0.1119
Location-Scale *t*	0.0246	0.0170	0.0214	0.0143	0.0156	0.0255
Logistic	0.0295	0.1096	0.0954	0.0144	0.3260	0.2276
Cauchy	0.0537	0.0346	0.0328	0.1142	0.0350	0.0405

**Table 6:** Estimated parameter values of candidate distribution models for 3D attitude jitter (estimated from the full steady cruise dataset, N= 707,823).

Distribution	Parameters	Pitch	Yaw	Roll
Normal	Location μ (°)	−0.0933	0.4163	−0.3235
	Standard deviation σ (°)	2.4972	5.3983	3.3328
Laplace	Location μ (°)	−0.0291	0.0615	−0.0512
	Scale parameter *b* (°)	1.8405	2.4101	1.8356
Location-Scale *t*	Shape (d.o.f.) ν	5.0315	1.4234	1.4242
	Location μ (°)	−0.0550	0.0273	−0.0286
	Scale parameter σ (°)	1.9823	1.2256	1.0257
Logistic	Location μ (°)	−0.0625	0.1214	−0.1294
	Scale parameter *s* (°)	1.3521	1.8169	1.3878
Cauchy	Location $x_{0}$ (°)	−0.0174	0.0248	−0.0238
	Scale parameter γ (°)	1.2474	1.0575	0.8738

**Table 7:** Fixed-wing UAV link characteristics and robustness design requirements.

Platform/Channel Characteristic	System Constraint	System Design
Platform Jitter	Pointing loss within power margin	HPHA > $5^{\circ }$, Receiver FOV > $10^{\circ }$
SWaP Constraints	Payload weight < 5 kg	Single OWC components and data processor both weigh 600 g
Communication Distance	Link power margin covering > 50 m	Maximum target range > 100 m@50 Mbps
Transmission Rate	Video stream requirement ≈ 8 Mbps	System maximum transmission rate 50 Mbps
Formations	Link rebuilding during maneuvers	Flight state feedforward compensation and rate-adaptive feedback protection

**Table 8:** Key performance parameters of the NIR-OWC system [26].

Parameter	Value
Maximum Error-Free Distance (MEFD)	197 m
HPHA (Yaw/Pitch)	6.47°/7.47°
Field of View (FOV)	14.36°
Bit Rate	100 Mbps
Output Optical Power	≈4 W
Operating Wavelength	940 nm

**Table 9:** Monte Carlo simulation configuration and initialization parameters.

Iterations *N*	Rate	$\sigma_{p}^{2}$	$\sigma_{v}^{2}$	$\sigma_{a}^{2}$	$\sigma_{p,0}^{2}$	$\sigma_{v,0}^{2}$
1000	10 Hz	$5.66\times 10^{-4}$ $m^{2}$	$3.9\times 10^{-5}$ (m/s)^2^	4.9877 (m/$s^{2}$)^2^	$1.707\times 10^{-3}$ $m^{2}$	$1.907\times 10^{-3}$ (m/s)^2^

**Table 10:** Overall system parameters of the fixed-wing UAV OWC scheme.

Parameter	Value
Total Operating Power	70 W
Operating Center Wavelength	940 nm
Achievable Data Rate	1–50 Mbps
Number of Concurrent OWC Links	3
Gimbal Coverage Range (Pitch/Yaw)	−$30^{\circ }$ to $90^{\circ }$/$\pm 60^{\circ }$
Transceiver Weight/Dimensions	600 g/160×140×100 ${\mathrm{mm}}^{3}$
Controller Weight/Dimensions	600 g/230×170×70 ${\mathrm{mm}}^{3}$

## Data Availability

The flight attitude dataset supporting the conclusions of this article is available at https://github.com/LiWentao0316/Fixed-Wing-UAV-Attitude-Dataset (accessed on 11 October 2026).

## Supplementary Materials

The following supporting information can be downloaded at: https://www.mdpi.com/article/10.3390/s26216896/s1, Visualization S1: Visual Demonstration of Two-UAV Video Transmission; Visualization S2: Visual Demonstration of Three-UAV Video Transmission.

## Abbreviations

The following abbreviations are used in this manuscript:

ACPC	Annular compound parabolic concentrator
APD	Avalanche photodiode
BER	Bit error rate
CDR	Clock and data recovery
CV	Constant velocity
DUV	Deep ultraviolet
EAM	Electro-absorption modulator
EKF	Extended Kalman filter
FEC	Forward error correction
FER	Frame error rate
FET	Field-effect transistor
FOV	Field of view
FPGA	Field-programmable gate array
FSO	Free-space optical
GPS	Global Positioning System
HAP	High-altitude platform
HPHA	Half-power half-angle
HTP	Hexagonal truncated pyramid
H–V	Hufnagel–Valley
IEC	International Electrotechnical Commission
IM/DD	Intensity modulation with direct detection
KL	Kullback–Leibler
KS	Kolmogorov–Smirnov
LD	Laser diode
LED	Light-emitting diode
LOS/LoS	Line of sight
LVDS	Low-voltage differential signaling
MEFD	Maximum error-free distance
MLE	Maximum likelihood estimation
MRR	Modulating retro-reflector
NIR	Near infrared
NRZ	Non-return-to-zero
OOK	On-off keying
OWC	Optical wireless communication
PDF	Probability density function
RF	Radio frequency
RMS	Root mean square
RS	Reed–Solomon
RSS	Root sum square
Rx	Receiver
Si APD	Silicon avalanche photodiode
SI	Scintillation index
SNR	Signal-to-noise ratio
STAR-RIS	Simultaneously transmitting and reflecting reconfigurable intelligent surface
SWaP	Size, weight, and power
TIA	Transimpedance amplifier
Tx	Transmitter
UAV	Unmanned aerial vehicle
UV	Ultraviolet
VCSEL	Vertical-cavity surface-emitting laser
VTOL	Vertical takeoff and landing

## Appendix A. Derivation of the PDF of the RSS of Two Independent Location-Scale *t*-Distribution Variables

Let *X* and *Y* be two independent location-scale *t*-distribution random variables:

(A1) $$\begin{array}{cc} f_{X}\left(x\right) & =\frac{\Gamma \left(\frac{v_{x}+1}{2}\right)}{\sqrt{\pi v_{x}}\tau_{x}\Gamma \left(\frac{v_{x}}{2}\right)}{\left(1+\frac{{\left(x-\mu_{x}\right)}^{2}}{v_{x}\tau_{x}^{2}}\right)}^{-\frac{v_{x}+1}{2}} \end{array}$$

(A2) $$\begin{array}{cc} f_{Y}\left(y\right) & =\frac{\Gamma \left(\frac{v_{y}+1}{2}\right)}{\sqrt{\pi v_{y}}\tau_{y}\Gamma \left(\frac{v_{y}}{2}\right)}{\left(1+\frac{{\left(y-\mu_{y}\right)}^{2}}{v_{y}\tau_{y}^{2}}\right)}^{-\frac{v_{y}+1}{2}} \end{array}$$

Assuming zero mean $\mu_{x}=\mu_{y}=0$, we look for the PDF of $R=\sqrt{X^{2}+Y^{2}}$. Transforming to polar coordinates:

(A3) x=rcosθ,y=rsinθ

The PDF of *R* is:

(A4) $$f_{R}\left(r\right)=\int_{0}^{2\pi }f_{X,Y}\left(x\left(r,\theta \right),y\left(r,\theta \right)\right)\left|\frac{\partial (x,y)}{\partial (r,\theta )}\right|d\theta$$

Since *X* and *Y* are independent:

(A5) $$f_{R}\left(r\right)=\int_{0}^{2\pi }f_{X}\left(r\cos\theta \right)f_{Y}\left(r\sin\theta \right)rd\theta$$

Due to symmetry, the integration range is reduced to [0,π/2]:

(A6) $$f_{R}\left(r\right)=\frac{4r\Gamma \left(\frac{v_{x}+1}{2}\right)\Gamma \left(\frac{v_{y}+1}{2}\right)}{\pi \sigma_{x}\sigma_{y}\sqrt{v_{x}v_{y}}\Gamma \left(\frac{v_{x}}{2}\right)\Gamma \left(\frac{v_{y}}{2}\right)}\int_{0}^{\pi /2}{\left(1+\frac{r^{2}\cos^{2}\theta }{v_{x}\sigma_{x}^{2}}\right)}^{-\frac{v_{x}+1}{2}}{\left(1+\frac{r^{2}\sin^{2}\theta }{v_{y}\sigma_{y}^{2}}\right)}^{-\frac{v_{y}+1}{2}}d\theta$$

Setting $t=\sin^{2}\theta$, $d_{1}=\frac{r^{2}}{v_{x}\sigma_{x}^{2}}$, and $d_{2}=\frac{r^{2}}{v_{y}\sigma_{y}^{2}}$, the integral is:

(A7) $$\begin{array}{cc} f_{R}\left(r\right)= & \frac{2r{\left(1+d_{1}\right)}^{-\frac{v_{x}+1}{2}}\Gamma \left(\frac{v_{x}+1}{2}\right)\Gamma \left(\frac{v_{y}+1}{2}\right)}{\pi \sigma_{x}\sigma_{y}\sqrt{v_{x}v_{y}}\Gamma \left(\frac{v_{x}}{2}\right)\Gamma \left(\frac{v_{y}}{2}\right)}\\ \; & \times \int_{0}^{1}t^{-1/2}{\left(1-t\right)}^{-1/2}{\left(1-\frac{d_{1}}{1+d_{1}}t\right)}^{-\frac{v_{x}+1}{2}}{\left(1+d_{2}t\right)}^{-\frac{v_{y}+1}{2}}dt \end{array}$$

Using the integral representation of the Appell $F_{1}$ function:

(A8) $$F_{1}\left(a;b,b^{\prime };c;x,y\right)=\frac{\Gamma (c)}{\Gamma (a)\Gamma (c-a)}\int_{0}^{1}u^{a-1}{\left(1-u\right)}^{c-a-1}{\left(1-ux\right)}^{-b}{\left(1-uy\right)}^{-b^{\prime }}du$$

We set a=1/2, $b=\frac{v_{x}+1}{2}$, $b^{\prime }=\frac{v_{y}+1}{2}$, c=1. The closed-form PDF of *R* is:

(A9) $$f_{R}\left(r\right)=EF_{1}\left(\frac{1}{2};\frac{v_{x}+1}{2},\frac{v_{y}+1}{2};1;\frac{r^{2}}{v_{x}\sigma_{x}^{2}+r^{2}},-\frac{r^{2}}{v_{y}\sigma_{y}^{2}}\right)$$

where

(A10) $$E=\frac{2r{\left(1+\frac{r^{2}}{v_{x}\sigma_{x}^{2}}\right)}^{-\frac{v_{x}+1}{2}}\Gamma \left(\frac{v_{x}+1}{2}\right)\Gamma \left(\frac{v_{y}+1}{2}\right)}{\sigma_{x}\sigma_{y}\sqrt{v_{x}v_{y}}\Gamma \left(\frac{v_{x}}{2}\right)\Gamma \left(\frac{v_{y}}{2}\right)}$$

## Appendix B. Proof of Convergence and Truncation Error Bounds of the Average BER Series

The overall BER includes $P_{\mathrm{out}}/2$; the convergence proof and truncation bound concern only the in-boundary series:

(A11) $${\bar{P}}_{e}=\frac{1}{2}P_{\mathrm{out}}+\frac{B{\left(\alpha \beta \right)}^{(\alpha +\beta )/2}}{\Gamma (\alpha )\Gamma (\beta )}\int_{1/A_{0}}^{u_{\max}}u^{(\alpha +\beta )/2-1}\sum_{m=0}^{\infty }\sum_{n=0}^{\infty }\left(\Phi \left(m,n\right)g_{m,n}\left(1/u\right)M\left(u\right)\right)du$$

Since the remaining factors, including $u^{(\alpha +\beta )/2-1}M\left(u\right)$, are continuous and integrable over the finite interval $\left[1/A_{0},u_{\max}\right]$, and the Appell series is uniformly and absolutely convergent within the domain where the arguments satisfy $x\left(u\right)\leq r_{1}<1$ and $y\left(u\right)\leq r_{2}<1$, Fubini’s theorem can be applied to swap the order of integration and summation.

We define the terms of the double series as follows:

(A12) $$U_{m,n}=\frac{B{\left(\alpha \beta \right)}^{(\alpha +\beta )/2}}{\Gamma (\alpha )\Gamma (\beta )}\Phi \left(m,n\right)I_{m,n}$$

where

(A13) $$I_{m,n}=\int_{1/A_{0}}^{u_{\max}}W\left(u\right){\left[x\left(u\right)\right]}^{m}{\left[y\left(u\right)\right]}^{n}du$$

and the common weight function W(u) is defined as follows:

(A14) $$W\left(u\right)=\frac{u^{(\alpha +\beta )/2-1}M\left(u\right)}{{\left(1+C\ln(A_{0}u)\right)}^{(v_{x}+1)/2}{\left(1+D\ln(A_{0}u)\right)}^{1/2}}$$

In terms of the reciprocal geometric attenuation variable $u=1/h_{g}\in \left[1/A_{0},u_{\max}\right]$, the transformed arguments $x\left(u\right)=x\left(h_{g}=1/u\right)$ and $y\left(u\right)=y\left(h_{g}=1/u\right)$ of the Appell series from Equation (16) are expressed as follows:

(A15) $$x\left(u\right)=1-\frac{1}{\left(1+C\ln(A_{0}u)\right)\left(1+D\ln(A_{0}u)\right)},\;y\left(u\right)=\frac{D\ln(A_{0}u)}{1+D\ln(A_{0}u)}$$

where the constants $C=\frac{\tan^{2}\left(\theta_{\mathrm{HPHA}}\right)}{v_{x}\sigma_{x}^{2}\ln2}$ and $D=\frac{\tan^{2}\left(\theta_{\mathrm{HPHA}}\right)}{v_{y}\sigma_{y}^{2}\ln2}$ are channel parameters determined by the transmitter beam divergence $\theta_{\mathrm{HPHA}}$, platform attitude jitter scales $\sigma_{x},\sigma_{y}$, and shape parameters $v_{x},v_{y}$. Because $\ln(A_{0}u)\geq 0$ for $u\geq 1/A_{0}$ with positive constants C,D>0, both x(u) and y(u) are strictly monotonically increasing functions of *u*. Over the operational integration interval $u\in [1/A_{0},u_{\max}]$, the upper limit $u_{\max}=1/h_{g,\min}=\frac{1}{A_{0}}\exp\left(\frac{\ln2\cdot \theta_{\max}^{2}}{\tan^{2}\left(\theta_{\mathrm{HPHA}}\right)}\right)$ is physically dictated by the maximum operational angular pointing misalignment $\theta_{\max}$ (constrained by the transceiver tracking envelope or receiver FOV). Consequently, the logarithmic products decouple directly into:

(A16) $$C\ln\left(A_{0}u_{\max}\right)=\frac{\theta_{\max}^{2}}{v_{x}\sigma_{x}^{2}},\;D\ln\left(A_{0}u_{\max}\right)=\frac{\theta_{\max}^{2}}{v_{y}\sigma_{y}^{2}}$$

The bounds $r_{1}$ and $r_{2}$ on the Appell series arguments are therefore explicitly linked to the physical platform and channel parameters as follows:

(A17) $$\begin{array}{cc} r_{1} & ≜\max_{u\in [1/A_{0},u_{\max}]}x\left(u\right)=x\left(u_{\max}\right)=1-\frac{1}{\left(1+\frac{\theta_{\max}^{2}}{v_{x}\sigma_{x}^{2}}\right)\left(1+\frac{\theta_{\max}^{2}}{v_{y}\sigma_{y}^{2}}\right)}\in \left(0,1\right) \end{array}$$

(A18) $$\begin{array}{cc} r_{2} & ≜\max_{u\in [1/A_{0},u_{\max}]}y\left(u\right)=y\left(u_{\max}\right)=\frac{\theta_{\max}^{2}}{v_{y}\sigma_{y}^{2}+\theta_{\max}^{2}}\in \left(0,1\right) \end{array}$$

Since $\theta_{\max}<\infty$, $\sigma_{x},\sigma_{y}>0$, and $v_{x},v_{y}>0$, we have $1+\frac{\theta_{\max}^{2}}{v_{x}\sigma_{x}^{2}}>1$, which strictly guarantees $0\leq r_{2}<r_{1}<1$. The joint convergence bound $r_{3}$ is thus explicitly given by:

(A19) $$r_{3}≜\max\left(r_{1},r_{2}\right)=r_{1}=1-\frac{1}{\left(1+\frac{\theta_{\max}^{2}}{v_{x}\sigma_{x}^{2}}\right)\left(1+\frac{\theta_{\max}^{2}}{v_{y}\sigma_{y}^{2}}\right)}<1$$

Because W(u) is continuous on the compact interval $\left[1/A_{0},u_{\max}\right]$, its absolute integral $C_{0}=\int_{1/A_{0}}^{u_{\max}}\left|W\left(u\right)\right|du$ is finite. Thus, $I_{m,n}$ can be bounded by:

(A20) $$|I_{m,n}|\leq C_{0}r_{1}^{m}r_{2}^{n}\leq C_{0}r_{3}^{m+n}$$

For shape parameters $v_{x}<2$ (which holds for our experimental flight attitude data with $v_{x}\approx 1.2–1.8$), the Gamma-function ratios in Φ(m,n) are bounded according to the Stirling/Tricomi-Erdélyi asymptotics. Hence, there exists a constant $C_{2}>0$, independent of *m* and *n*, such that:

(A21) $$\left|\Phi \left(m,n\right)\right|\leq C_{2}$$

We obtain the upper bound for the terms:

(A22) $$|U_{m,n}|\leq M_{m,n}^{*}=C_{3}r_{3}^{m+n}$$

The series $\sum \sum M_{m,n}^{*}$ is geometric and absolutely convergent since $r_{3}<1$. The truncation error E(M,N) after truncating the double series to *M* and *N* terms is:

(A23) $$\begin{array}{cc} E(M,N) & \leq \sum_{m=M+1}^{\infty }\sum_{n=0}^{\infty }|U_{m,n}|+\sum_{m=0}^{M}\sum_{n=N+1}^{\infty }\left|U_{m,n}\right|\\ \; & \leq \frac{C_{3}}{{\left(1-r_{3}\right)}^{2}}\left(r_{3}^{M+1}+r_{3}^{N+1}\right)\leq \frac{2C_{3}}{{\left(1-r_{3}\right)}^{2}}r_{3}^{\min(M,N)+1} \end{array}$$

This completes the proof of absolute convergence and gives the exponential truncation error bound.
